# Implications of zonal architecture on differential gene expression profiling and altered pathway expressions in mandibular condylar cartilage

**DOI:** 10.1038/s41598-021-96071-7

**Published:** 2021-08-19

**Authors:** Aisha M. Basudan, Mohammad Azhar Aziz, Yanqi Yang

**Affiliations:** 1 Division of Orthodontics, Dental Services Department, KAMC/KAIMRC/King Saud bin Abdulaziz University for Health Sciences (KSAU-HS), Ministry of National Guard-Health Affairs (MNGHA), Riyadh, 11426 Saudi Arabia; 2 King Abdullah International Medical Research Center (KAIMRC)/King Saud bin Abdulaziz University for Health Sciences (KSAU-HS), Colorectal Cancer Research Program, MNGHA, Riyadh, 11426 Saudi Arabia; 3 Division of Paediatric Dentistry and Orthodontics, Faculty of Dentistry, The University of Hong Kong, 34 Hospital Road, Hong Kong, SAR China

**Keywords:** Temporomandibular disorders, Cartilage development, Microarray analysis

## Abstract

Mandibular condylar cartilage (MCC) is a multi-zonal heterogeneous fibrocartilage containing different types of cells, but the factors/mechanisms governing the phenotypic transition across the zones have not been fully understood. The reliability of molecular studies heavily rely on the procurement of pure cell populations from the heterogeneous tissue. We used a combined laser-capture microdissection and microarray analysis approach which allowed identification of differential zone-specific gene expression profiling and altered pathways in the MCC of 5-week-old rats. The bioinformatics analysis demonstrated that the MCC cells clearly exhibited distinguishable phenotypes from the articular chondrocytes. Additionally, a set of genes has been determined as potential markers to identify each MCC zone individually; Crab1 gene showed the highest enrichment while Clec3a was the most downregulated gene at the superficial layer, which consists of fibrous (FZ) and proliferative zones (PZ). Ingenuity Pathway Analysis revealed numerous altered signaling pathways; Leukocyte extravasation signaling pathway was predicted to be activated at all MCC zones, in particular mature and hypertrophic chondrocytes zones (MZ&HZ), when compared with femoral condylar cartilage (FCC). Whereas Superpathway of Cholesterol Biosynthesis showed predicted activation in both FZ and PZ as compared with deep MCC zones and FCC. Determining novel zone-specific differences of large group of potential genes, upstream regulators and pathways in healthy MCC would improve our understanding of molecular mechanisms on regional (zonal) basis, and provide new insights for future therapeutic strategies.

## Introduction

Temporomandibular joint (TMJ) is described as a synovial sliding-ginglymoid, load-bearing joint where the mandibular condyle is capable of not only rotary (hinge) but also translatory (sliding) movements during the daily tasks including chewing, speaking, swallowing, and yawning^1–3^. Temporomandibular joint disorder (TMD) is a class of degenerative musculoskeletal conditions manifested as deformities in the morphology and function of the TMJ^3,4^. It includes abnormal position and/or structure of the TMJ disc and dysfunction of the associated musculature of the face (orofacial pain)^3,5^. TMD together with other conditions such as arthritis, congenital anomalies, and injuries/truama to the TMJ are significant causes of morbidity that can negatively affect the quality of life of human beings.

Clinical management starts with noninvasive treatment modalities (physical therapy, occlusal splints, and prescription of pharmacologic agents), but for some patients other minimally invasive strategies (injections of sodium hyaluronate and/or corticosteroids, arthrocentesis, and arthroscopy) are considered^3^. For those patients who show no improvement with the nonsurgical treatment modalities, open joint surgery may be carried out for discectomy, reshaping or reconstructing the articulating surfaces, and total joint replacement which is the most invasive option^3^. Recently, there is an emphasis to apply cell-based regenerative therapies^5,6^, but one common problem is the unfamiliarity with and lack of thorough understanding of native tissue characteristics^3^.

The advent of microarray technology has enabled the researchers to analyze the expression of thousands of genes simultaneously in a single experiment^7^, and provided a huge amount of information about gene expression for cells^8^. However, the reliability and validity of such molecular studies is totally dependent on the procurement of pure cell populations with relatively high abundance^9^. Hence, the two impediments are the heterogeneity of the native tissues and the abundance of the biomolecules extracted from cells. The larger the number of “contaminating/unwanted” cells upon procurement, the greater the chances of getting false results and inaccurate interpretation of the data^9^. Based on the histology, typical articular cartilage can be divided into three distinct layers: the superficial, middle, and deep zones, with variation in the morphology and density of the chondrocytes^10–12^. The mandibular condylar cartilage (MCC), a fibrocartilage layer that covers the mandibular condyle of TMJ, is also multizonal in structure but it is unique in terms of cell phenotypes. From the articular surface of MCC to the underlying bone, the following four zones are identifiable: fibrous (FZ), proliferative (PZ), mature (MZ), and hypertrophic (HZ) zones^13,14^. Cells have traditionally been harvested from MCC by mincing the tissue with a scalpel, followed by enzymatic digestion; consequently, the outcome includes various cell types from the four different zones^15^. Gross sampling may conceal the individual gene expression profiling of zone-specific cell populations^16^. On the other hand, Laser-Capture microdissection (LCM) technology, allows precise procurement of cells of interest from a heterogeneous tissue in a relatively rapid and practical manner^17^. Moreover, DNA (Deoxyribonucleic acid), RNA (Ribonucleic acid), and proteins can be extracted from the homogenous isolates of cell population^18^, and that could be followed by an array of analytical applications, which allows reliable studying of in vivo genomic and proteomic profiling of the tissue of concern. Generating microarray data from LCM samples is feasible^9^, however, such a combination should be capable of coping with several challenges^19,20^.

Beside its unique histomorphological zonal organization, MCC is distinguished from growth and articular cartilages in the embryonic origin, biochemical and biomechanical properties^14,21–23^. Functionally, MCC has a dual role; one as an articular fibrocartilage responsible for load distribution and disc articulation, and the other one as a major site responsible for mandibular growth^24^. Additional importance of the MCC stems from its role in life-long bone remodeling process. In light of these distinctive features, it would not be surprising if the molecular and genetic regulation of the biological processes of MCC were different from those of other articular hyaline cartilages and epiphyseal growth cartilages. In addition, the MCC is a multi-zonal fibrocartilage containing different types of cells which are well characterized histomorphologically^6,25^ but the factors governing their morphological transition across the zones have not been fully understood. Therefore, it is logical to speculate that unique genetic profiles in vivo might exist across the four MCC zones.

A crucial prerequisite for functional replacement of affected/diseased MCC is to generate accurate knowledge at cellular and molecular level. Combined LCM and MAA (microarray analysis) enable large-scale in situ studies that could clarify many hidden or masked diagnostic and therapeutic aspects which were not previously identified^16^. However, very little is reported on the regional or zonal genetic profiling and molecular phenotypes of MCC cells in the literature^6,26,27^. The aim of our study is to perform a comprehensive gene expression profile analysis with a specific focus on pathways analysis for each zone of the MCC from 5-week-old rats using LCM and MAA, and to formulate a well-supported hypothesis to identify genes which could potentially distinguish the cells of MCC zones from each other and from the articular chondrocytes.

## Materials and methods

### Preparation of LCM samples and RNA extraction

Animal use protocol was approved by the Committee on the Use of Live Animals in Teaching and Research of the University of Hong Kong (CULATR 2311-11), and the procedures were carried out in accordance with the institutional guidelines and in accordance with ARRIVE guidelines (https://arriveguidelines.org). Sprague–Dawley 5-week-old male rat (Rattus norvegicus) was sacrificed by intraperitoneal injection using 20% Dorminal (200 mg pentobarbital sodium, Alfasan, Woerden-Holland, Netherlands) with a dose of 100 mg per 100 g of body weight. MCC and FCC specimens were harvested, and then our optimized LCM protocol described earlier^16^ was applied to collect RNA from the four zones of MCC individually thereby four groups: FZ, PZ, MZ, and HZ were prepared (Supplementary Fig. 1a). Similarly LCM sample was prepared by microdissecting the chondrocytes from the middle and deep zones of FCC tissue (group C) as a control (Supplementary Fig. 1b). Briefly, the freshly dissected specimens were embedded in optimal cutting temperature (OCT), frozen on precooled isopentane (Sigma-Aldrich, USA). Frozen sections were cut at 7 μm thickness using a cryostat set to − 24 °C to − 30 °C, and were mounted on glass microscope slide (HistoBond + adhesive microscope slides, Marienfeld laboratory glassware, Germany). Slides were processed using Arcturus HistoGene LCM Frozen Section Staining Kit (CA, USA) was used according to the manufacturer’s instructions, but with a few modifications. The slides were individually placed onto the microscope platform of the Arcturus PixCell II Laser Capture Microdissection System (CA, USA). Under 10× objective, the unstained tissue sections provided adequate morphology to distinguish MCC zones. LCM was performed and the collected dissected cells were immediately lysed in 50 μL of lysis buffer (Arcturus PicoPure RNA Isolation Kit, Applied Biosystems, CA, USA) as recommended by the manufacturer. The cell lysate of 30 microdissected tissue sections from each zone were and pooled to create single samples, except for FZ group where the lysates of 60 microdissected sections were pooled. RNA was isolated from the pooled samples using Arcturus PicoPure RNA Isolation Kit (Applied Biosystems). To assess RNA quality, 1 μL was analyzed using RNA 6000 Pico kit and Bio-Analyzer 2100 (Agilent Biotechnologies). RNA integrity number (RIN) values were > 5 which is within the accepted range for LCM samples^28^.

### Amplification, labeling, fragmentation, and hybridization of RNA samples

Eleven µl of each sample was used as total RNA input material for a two-cycle linear amplification process in accordance to the protocol provided with the Arcturus RiboAmp HS Plus amplification Kit (Applied Biosystems, CA, USA) (https://tools.lifetechnologies.com/content/sfs/manuals/cms_085206.pdf). After the first strand cDNA synthesis reaction of the first amplification round, 2 μl of each sample was removed to assess the integrity of the starting mRNA by evaluating the 3′/m ratio of GAPDH and β-Actin housekeeping genes via the qRT-PCR assay as described previously^16^. Concurrently, RNA control provided with the kit was also amplified to calculate the amplification efficiency by dividing the RNA yield after amplification over the initial RNA input (500 pg). Following the amplification process, quality control measurements were employed to determine the mRNA transcript length using Agilent 2100 Bioanalyzer with a total RNA 6000 Nano-lab-Chip (Agilent technologies, CA, USA). RNA was quantified using a Nanodrop 2000c spectrophotometer (Thermo Scientific) and assessed for purity where a ratio of two optical densities; A260 and A280 (OD 260/280) was calculated. Labeling of aRNA transcripts was performed using the Arcturus Turbo Labeling Biotin Reagent Kit (Applied Biosystems, Amsterdam, The Netherlands) and according to the manufacturer’s instructions (https://tools.lifetechnologies.com/content/sfs/manuals/cms_085525.pdf). After quantification, 16.8 µg of the labeled aRNA samples were hydrolyzed in a fragmentation buffer (5×) solution of the GeneChip 3′ IVT Express Kit (Affymetrix, Santa Clara, CA, USA). The protocol followed was in accordance with the Arcturus Turbo Labeling Kit with Biotin manufacturer’s instructions (https://tools.lifetechnologies.com/content/sfs/manuals/cms_085525.pdf). Fragment size of the products was confirmed using the Agilent 2100 Bioanalyzer. Fragmented and labeled aRNA samples (12.5 μg in 25 μl each) were sent to the Center for Genomic Sciences/The University of Hong Kong (CGS/HKU) facility where microarray hybridization, scanning and image analysis procedures were carried out using GeneChip Rat Genome 230 2.0 Array (Affymetrix, Santa Clara, CA, USA), which contained 31,099 probesets, according to the Affymetrix GeneChip Expression Analysis Technical Manual (http://jaxservices.jax.org/Affymetrix_Gene_expression_manual430.pdf). Each probe set in the array is represented by 11 pairs consisting of 25mer oligonucleotides, and each pair includes a perfect match oligo (complementary to the aRNA target) and a mismatch oligo to calculate the nonspecific and background hybridization.

Microarray data have been submitted to the National Centre for Biotechnology Information (NCBI) and was deposited in gene expression omnibus (GEO) (accession number: GSE162823).

### Microarray data analysis

Following hybridization, the array was washed, stained, and scanned, and then resultant data were analyzed. Microarray Suite version 5 (MAS5.0 Affymetrix, Santa Clara, CA, USA) software enables both probe set summarization, as well as initial quality examination of data. MAS5.0 probe summarization algorithms included: background correction, probe summarization (to convert probe level values to probeset expression values), and normalization. Background correction was achieved by subtracting the signal of the nonspecific binding (mismatch) probe from that of the perfect match probe for each of the 11 pairs of a probe set, then the 11 intensities were condensed to one value per gene (probe set). To identify the differentially expressed genes among the MCC zones as well as FCC, MAA data were further analyzed using GeneSpring GX version 12 software (Agilent Technologies Inc., Santa Clara, CA, USA) where the Biological Significance Analysis workflow was selected. In this workflow type, default parameters for data processing are applied, and according to the RMA (Robust Multichip Averaging) normalization approach, absolute expression values were baselined to a median of all samples expression value, normalized using the quantile scheme, and then transformed into log_2_ based value of relative intensity for each probe set. The data sets were then filtered by removing the lowest 20% of intensity values, leaving data from 26,121 probe sets. For fold change (FC) analysis, the ratio between the normalized intensities of a probe set belonging to two groups of the samples is calculated. The default cut off for the FC, which is *log*_*2*_ value of 2, was used to identify genes with expression ratios outside of this value in any of the ten pair conditions (FZ/C, PZ/C, MZ/C, HZ/C, FZ/PZ, FZ/MZ, FZ/HZ, PZ/MZ, PZ/HZ, and MZ/HZ). Any fold-change value that is less than one was replaced by the negative of its inverse.

To asses the biological functions of the differentially expressed genes at FC >  ± 1.4 (a total of 4634 genes, Supplementary Table  1), data were analyzed through the use of IPA (QIAGEN Inc., https://www.qiagenbioinformatics.com/products/ingenuitypathway-analysis)^29^. Core analysis that identifies top canonical pathways, upstream regulators, biological and diseases function, and toxicity function for each pairwise comparisons was generated. The significance of IPA core analysis was measured in two ways: (1) a Fischer’s exact test was used to calculate a p-value determining the probability that the association between the differentially expressed genes and the canonical pathway is explained by chance alone; (2) Z-score to provide predictions (increased function/ decreased function/no effect) about upstream or downstream processes.

### Quantitative real-time PCR (qRT-PCR)

RNA samples were used as a template to synthesize first-strand cDNA according to the manufacturer's instructions using Superscript III Reverse Transcriptase (Invitrogen, CA, USA) and oligo(dT)_12–18_ (Invitrogen, CA, USA). Ten μL of LCM-RNA was used per 20 μL cDNA synthesis reaction using Veriti 96 well thermal cycler (Applied Biosystems, CA, USA). Ten genes were randomly selected from the list of differentially expressed genes of the microarray data namely; Car9, Cmtm5, Ctsz, Drd4, Dusp27, Fam180a, Gdf10, Itgbl1, RGD 1311447, and Ucma. qRT-PCR was performed using a Step One Plus RT-PCR system (Applied Biosystems, CA, USA) and Power SYBR^®^ Green PCR master mix (Applied Biosystems, Warrington, UK). Relative quantities for the tested genes were determined utilizing the corresponding standard curves generated in the same experiment and *GAPDH* as the endogenous control as previously described^16^.

## Results

### Gene expression signature

We were able to create zone-specific gene expression profiles of MCC by combining LCM and MAA technologies.

#### High and low absolute expression levels of genes

The top 10 genes with greatest/least absolute expression levels in each group are listed in (Table 1, Supplementary Figs. 2–11). Most of the highly expressed genes in group C had similar profiles that show high abundance in the FCC tissue with much lower expressed values in all MCC zones. The most interesting finding among the profiles of genes with high/low absolute expression values was the distinctive profiles of the 10 genes that showed the least abundance in PZ. These genes were uniquely downregulated at PZ but with much higher abundance levels in all other zones. In addition to this unizonal characteristic, analysis of the absolute expression values also revealed a bizonal pattern. Considerable overlap was found between FZ and PZ, for instance, Crapb1, Tnmd, Dpt, Bcl11b, Plxdc1, P4ha, Aspn, Hs3st6, Pcdh20 and Fndc1 genes tend to be highly expressed in both FZ and PZ while reduced in other zones. Similarly, the top genes identified as the greatest (or least) expressed at MZ were also increased (or reduced) for HZ. In the subset of the lowest 10 genes expressed at HZ, Fndc1 was found to have a profile different from the other 9 members where it was markedly high at both FZ & PZ zones and extremely low at HZ. For the remaining 9 genes, the expression levels at MZ were close to those of HZ especially for Angptl1, Col14a1, and Cpxm2.

**Table 1:** The top ten annotated genes with the greatest/lowest absolute expression levels in the FCC and four zones of MCC tissues.

Groups	Genes with the greatest absolute expression levels	Genes with the lowest absolute expression levels
C (femoral condylar cartilage)	**Mfi2** (antigen p97 (melanoma associated),	**Dusp27** (dual specificity phosphatase 27 putative)
	**Mia** (melanoma inhibitory activity)	**LOC685277** (similar to liver-specific bHLH-Zip transcription factor)
	**RGD1566401** (Similar to GTL2, imprinted maternally expressed untranslated)	**Robo2** (roundabout, axon guidance receptor, homolog 2)
	**Gdf10** (growth differentiation factor 10)	**Lox** (lysyl oxide)
	**Hoxc9** (homeobox C9)	**Serpinf1** (serine or cysteine peptidase inhibitor, clade B)
	**Chad** (chondroadherin)	**Dlx1** (distal-less homeobox 1)
	**Hoxc10** (homeobox C10)	**Slfn3** (schlafen 3)
	**A1i3/Mug1** (alpha-1-inhibitor III, murinoglobulin 1)	**Tes** (testis derived transcript)
	**Ptgds** (prostaglandin D2 synthase brain)	**Rasal2** (RAS protein activator like2)
	**Atp1a2** (ATPase, Na+/K+ transporting, alpha 2 polypeptide)	**Fam25a** (family with sequence similarity 25, member A)
FZ (fibrous zone)	**Tnmd** (tenomodulin)	**Clec3a** (C-type lectin domain family, member a)
	**Crabp1** (cellular retinoic acid binding protein 1)	**Col9a1** (collagen, type IX, alpha 1)
	**Dpt** (dermatopontin)	**Foxa2** (forkhead box A2)
	**Bcl11b** (B-cell CLL/lymphoma 11B (zinc finger protein)	**Hils1** (histone linker H1 domain, spermatid-specific 1)
	**Igfbp6** (insulin-like growth factor binding protein 6)	**Col9a3** (collagen, type IX, alpha 3)
	**Plxdc1** (plexin domaincontaining1	**Matn3** (matrilin 3)
	**Fndc1** (fibronectin type III domain containing 1)	**Cmtm5** (CKLF-like Marvel transmembrane domain containing5
	**P4ha3** (procollagen-proline, 2-oxoglutarate 4-dioxygenase (proline 4-hydroxylase), alpha)	**Mcoln2** (mucolipin2)
	**Mfap4** (microfibrillar-associated protein 4)	**Col9a2** (collagen, type IX, alpha 2)
	**Crabp2** (cellular retinoic acid binding protein 2)	**Col10a1** (collagen, type X, alpha 1)
PZ (proliferative zone)	**Bcl11b** (B-cell CLL/lymphoma 11B zinc finger protein)	**Ubl5** (ubiguitin-like5)
	**P4ha3** (pro collagen-proline, 2-oxoglutarate)	**Ccl9** (chemokine C–C motifligand9
	**Plxdc1** (plexin domaincontaining1	**Tp53** (tumor protein p53)
	**Aspn** (asporin)	**Hsp90ab1** (heat shock protein 90 kDa alpha cytosolic class)
	**Tnmd** (tenomodulin)	**Aplp2** (amyloid beta A4 precursor-like protein 2)
	**Dpt** (dermatopontin)	**Col4a2** (collagen, type IV, alpha 2)
	**Hs3st6** (heparan sulfate glucosamine)	**Fgl2** (fibrinogen-like2)
	**LOC688502** (similar to protein arginine N-methyltransferase	**Nab2** (Ngfi-A binding protein 2)
	**Pcdh20** (protocadherin 20)	**Kif13a** (kinesin family member 13A)
	**Rgs7bp** (regulator of G-protein signaling 7 binding protein)	**Atp11b** (ATPase, class VI, type 11B)
MZ (mature zone)	**Serpinb10** (serine or cysteine peptidase inhibitor, clade B)	**Angptl1** (angiopoietin-like 1)
	**Mmrn1** (mulimerin 1)	**Cpxm2** (carboxypeptidase X M14 family, member 2)
	**Plek** (pleckstrin)	**Col14a1** (collagen, type XIV, alpha 1)
	**Pf4** (platelet factor 4)	**Hmcn1** (hemicentin 1)
	**Nubp2** (nucleotide binding protein 2)	**Itgbl1** (integrin, beta-like 1)
	**Sstr2** (somatostatin receptor 2)	**Aoc3** (amine oxidase, copper containing 3)
	**Lect1** (leukocyte cell derived chemotaxin 1)	**Tspan2** (tetraspanin2)
	**Treml1** (triggering receptor expressed on myeloid)	**Fibin** (fin bud initiation factor homolog)
	**RGD1564318** (similar to immunoglobulin light chain variable)	**Pon3** (Paraoxonase3)
	**Pla2g2a** (phospholipase A2, group IIA)	**Casr** (calcium-sensing receptor)
HZ (hypertrophic zone)	**Car1** (carbonic anhydrase 1)	**Car9** (carbonic anhydrase 9)
	**Slc4a1** (solute carrier family 4, anion exchanger)	**Angptl1** (angiopoietin-like 1)
	**RGD1564318** (similar to immunoglobulin light chain variable)	**Col14a1** (collagen, type XIV, alpha 1)
	**Pf4** (platelet factor 4)	**Matn2** (matrilin 2)
	**LOC100361706, LOC682411** (lambda-chain-C1-region-like	**Fam180a** (family with sequence similarity 180, member A)
	**Klf1** (Kruppel-like factor 1 erythroid)	**Fmod** (fibromodulin)
	**RGD1560020_predicted** (SIMILAR to Myb proto-oncogene protein C-myb), **Tal1** (T-cell acute lymphocytic leukemia 1)	**Cpxm2** (carboxypeptidase X M14 family, member 2)
	**Cd3g** (CD3 molecule, gamma polypeptide)	**Npas2** (neuronal PAS domain protein 2)
	**Cmah** (cytidine monophosphate-N-acetylneuraminic	**Fndc1** (fibronectin type III domain containing 1)
	**Ctse** (cathepsin)	**Ucma** (upper zone of growth plate and cartilage matrix associated)

#### Differential expression of genes by fold change analysis

The most commonly used application of MAA is identification of the differentially expressed genes rather than absolute quantification of RNA transcript abundance. Gene expression profile of each MCC zones was compared with group C (FCC tissue), in addition, MCC zones were compared against each other. Thereby, ten pairwise comparisons were established to assess the relative gene expressions; FZ versus C, PZ versus C, MZ versus C, HZ versus C, FZ versus PZ, FZ versus MZ, FZ versus HZ, PZ versus MZ, PZ versus HZ, and MZ versus HZ. Fold change (FC) analysis of a minimum *log*_*2*_ value of 2 in at least one of the 10 pairwise comparisons indicated that 2022 transcripts were differentially expressed (Supplementary Table 2). When MCC zones were compared with FCC (FZ/C, PZ/C, MZ/C, HZ/C comparisons), the total number of differentially expressed transcripts was 1670, of which 833 were downregulated in MCC zones relative to to FCC and 837 genes were upregulated in MCC (Supplementary Table 3). Comparisons of MCC zones with each other (FZ/PZ, FZ/MZ, FZ/HZ, PZ/MZ, PZ/HZ, and MZ/HZ comparisons) revealed 874 differentially expressed genes (Supplementary Table 4).

To focus on the strongly up- and down-regulated genes, only genes with ≥ 50 FC (or ≥ 5.64 *log*_*2*_ FC) were selected to compare MCC zones to FCC. The created gene subset consisted of 30 genes categorized either as transporters (Crabp1, Atp1a2), cytokines (Cmtm5), peptidases (Capn6), growth factor (Gdf10, Wisp3), transcription regulators (Hoxc9, Hoxc10), G-protein coupled receptors (Agtr2, Casr), enzymes (Ptgds), or others. The genes of this subset were extremely downregulated in MCC zones as compared to FCC except for Crabp1 (Table 2). This gene was among the highly expressed ones at FZ and PZ but with relatively reduced levels at MZ, HZ, and FCC (Table 1, Supplementary Figs. 4 and 6), indicating that Crabp1 upregulation could be a characteristic feature for FZ and PZ. Extreme downregulation of many genes (e.g. Lect1, RGD1311447, Mfi2, Mia, RGD1566401, Chad, Vit, Tpd52l1, Gdf10, A1i3/Mug1, Hoxc9, Agtr2, Hoxc10, and Ptgds) in MCC zones could be attributed to the greatly increased absolute expression of these genes in FCC tissue (Table 1, Supplementary Fig. 2). On the other side, Clec3a and Matn3 which are the most downregulated genes with − 8.92 and − 7.71 FC respectively, showed reduced absolute expression at FZ which might be a unique characteristic for this zone (Table 1, Supplementary Fig. 5).

**Table 2:** Top 30 differentially expressed genes that showed strong modulation in at least one of the four pairwise comparisons where each MCC zone was compared to FCC (the control, C) at ≥ 50 -fold change cut-off value (log_2_ 50 FC = 5.64, bold and italics indicate gene upregulation and downregulation respectively above the cut-off value).

Probe set ID	Entrez gene	Gene title	Gene symbol	Log_2_ Fold changes of pairwise comparisons with ≥ 50 FC^a^			
				FZ vs C	PZ vs C	MZ vs C	HZ vs C
1397360_at	365009	C-type lectin domain family 3, member a	Clec3a	*− 8.92*	− 3.02	− 2.76	*− 6.21*
1393943_at	313954	Matrilin 3	Matn3	*− 7.71*	− 4.44	− 3.32	− 4.44
1387164_at	81512	Leukocyte cell derived chemotaxin 1	Lect1	*− 7.52*	*− 5.71*	− 3.38	− 5.46
1393931_at	363276	LOC363276	RGD1311447	*− 7.46*	− 5.13	− 3.32	− 5.51
1372647_at	100363743	Proline arginine-rich end leucine-rich repeat protein-like	LOC100363743	− 1.86	− 3.91	− 5.28	*− 7.27*
1380270_at	288038	Antigen p97 (melanoma associated) identified by monoclonal antibodies 133.2 and 96.5	Mfi2	*− 7.22*	*− 6.47*	*− 6.47*	*− 7.19*
1391074_at	25061	Cellular retinoic acid binding protein 1	Crabp1	**7.21**	4.83	2.32	− 0.24
1369320_at	81510	Melanoma inhibitory activity	Mia	*− 7.21*	*− 6.40*	− 5.17	*− 7.09*
1383708_at	498564	Integrin, beta-like 1	Itgbl1	− 2.78	− 3.84	*− 6.75*	*− 6.84*
1377008_at	500717	Similar to GTL2, imprinted maternally expressed untranslated	RGD1566401	− 5.26	*− 6.12*	*− 6.76*	*− 6.77*
1388973_at	305104	Collagen, type IX, alpha 1	Col9a1	*− 6.66*	− 2.13	− 1.47	− 3.46
1368788_at	29195	Chondroadherin	Chad	− 5.15	*− 6.42*	*− 5.79*	*− 6.49*
1392832_at	679942	Angiopoietin-like 1	Angptl1	− 0.26	− 1.86	− 5.34	*− 6.46*
1387886_at	84400	Proline/arginine-rich end leucine-rich repeat protein	Prelp	− 2.13	− 3.67	− 4.83	*− 6.40*
1385682_at	313831	Vitrin	Vit	*− 6.36*	*− 6.33*	− 5.05	− 4.69
1382096_at	290214	CKLF-like MARVEL transmembrane domain containing 5	Cmtm5	*− 6.24*	− 3.10	− 2.06	− 4.87
1372626_at	689256	Tumor protein D52-like 1	Tpd52l1	*− 6.22*	*− 5.98*	− 3.73	− 5.18
1384202_at	288689	Tescalcin	Tesc	*− 6.17*	*− 6.07*	− 4.06	− 5.25
1368131_at	83685	Calpain 6	Capn6	− 2.29	− 2.64	− 3.42	*− 6.17*
1368459_at	79216	Growth differentiation factor 10	Gdf10	− 5.37	*− 6.12*	*− 5.95*	*− 5.94*
1378873_at	690026	Histone linker H1 domain, spermatid-specific 1	Hils1	*− 6.08*	− 1.85	− 1.07	− 4.46
1370027_a_at	297568///497794	Alpha-1-inhibitor III///murinoglobulin 1	A1i3///Mug1	*− 6.01*	*− 5.68*	− 5.45	*− 6.06*
1386911_at	24212	ATPase, Na+/K+ transporting, alpha 2 polypeptide	Atp1a2	− 5.61	− 5.61	*− 5.90*	*− 6.05*
1368978_at	64458	Stimulator of chondrogenesis 1	Scrg1	*− 6.05*	− 4.66	− 4.11	*− 5.88*
1380442_at	368178	Homeobox C9	Hoxc9	*− 6.01*	*− 5.81*	*− 5.82*	*− 5.82*
1397945_at	499461	WNT1 inducible signaling pathway protein 3	Wisp3	*− 5.96*	− 4.01	− 4.22	*− 5.95*
1398288_at	24182	Angiotensin II receptor, type 2	Agtr2	*− 5.77*	− 5.53	− 5.56	*− 5.92*
1369158_at	24247	Calcium-sensing receptor	Casr	− 3.37	− 3.59	*− 5.91*	− 5.41
1385113_at	315338	Homeo box C10	Hoxc10	*− 5.89*	*− 5.71*	*− 5.79*	*− 5.76*
1367851_at	25526	Prostaglandin D2 synthase (brain)	Ptgds	− 5.62	*− 5.71*	*− 5.67*	*− 5.67*

^a^For each gene in the pairwise comparison, there were 2 normalized intensity values (one for each group) representing the expression levels. The fold change (FC) value for the gene is calculated by dividing the larger value by the smaller one, then a positive sign is assigned if the gene is upregulated, in other words the gene expression value of the sample (the first group) is greater than the reference (the second group). On the other hand, downregulation of the gene (negative sign) indicates that the gene expression value of the sample is less than the reference.

Upon comparing MCC zones against each other, genes of the six pairwise comparisons were moderately modulated, unlike the strong modulation demonstrated above when MCC zones were compared to FCC. Additionally, the predominance of genes downregulation shown in Table 2 was not as evident in Table 3. In fact, FC analysis at ≥ 20 FC (or ≥ 4.32 *log*_*2*_ FC) cut-off value revealed 30 differentially expressed genes among MCC zones; 5 downregulated at FZ and 25 upregulated at FZ & PZ, and can be classified as transporters (Crabp1), kinases (Ephb3), peptidases (Cpxm2), growth factor (Igf2), transcription regulators (Bcl11b, Foxa2), G-protein coupled receptors (Mrgprf), and others. Table 3 also illustrates that 24 and 7 genes were upregulated in FZ/HZ and FZ/MZ comparisons respectively, similarly 10 genes showed upregulation at PZ relative to HZ. Such modulation finding at relatively high cut-off FC value would indicate the drastic differences between the phenotypes of corresponding cells of MCC zones, particularly the non-adjacent zones. In general, most of this subset genes were moderately upregulated at FZ and PZ and downregulated at MZ and HZ. For instance, the abundance of Crabp1, Fndc1, and Dpt genes was noticeably high at FZ and PZ, and relatively low at MZ and HZ, leading to extremely high modulations especially for FZ/HZ and PZ/HZ comparisons (Table 1). On the other hand, Clec3a, Col9a1, Hils1, Foxa2, and Matn3 genes, which showed the least absolute expression at FZ, were uniquely downregulated when comparing FZ to both PZ and MZ.

**Table 3:** Top 30 differentially expressed genes that showed strong modulation in at least one of the six pairwise comparisons where MCC zones were compared against each other at ≥ 20 -fold change cut-off value (log_2_ 20 FC = 4.32, bold and ilatlics indicate gene upregulation and downregulation respectively above the cut-off value).

Probe set ID	Entrez gene	Gene title	Gene symbol	Log_2_ Fold changes of pairwise comparisons with ≥ 20 FC^a^					
				FZ vs PZ	FZ vs MZ	FZ vs HZ	PZ vs MZ	PZ vs HZ	MZ vs HZ
1391074_at	25061	Cellular retinoic acid binding protein 1	Crabp1	2.38	**4.89**	**7.45**	2.51	**5.07**	2.56
1374726_at	308099	Fibronectin type III domain containing 1	Fndc1	0.84	4.06	**7.26**	3.22	**6.42**	3.20
1373947_at	289178	Dermatopontin	Dpt	2.67	**4.86**	**6.52**	2.20	3.86	1.66
1393452_at	313495	Carbonic anhydrase 9	Car9	1.67	3.11	**6.48**	1.43	**4.81**	3.37
1392832_at	679942	Angiopoietin-like 1	Angptl1	1.60	**5.08**	**6.20**	3.48	**4.60**	1.12
1376105_at	314981	Collagen, type XIV, alpha 1	Col14a1	1.60	**4.91**	**5.93**	3.32	**4.33**	1.01
1381504_at	306805	Asporin	Aspn	0.29	3.20	**5.87**	2.91	**5.59**	2.68
1368237_at	64104	Tenomodulin	Tnmd	2.43	**5.65**	**5.63**	3.22	3.20	− 0.02
1377086_at	294806	C1q and tumor necrosis factor related protein 3	C1qtnf3	− 0.07	2.36	**5.51**	2.43	**5.58**	3.15
1389306_at	299996	Matrilin 2	Matn2	1.15	2.82	**5.45**	1.68	4.30	2.63
1372647_at	100363743	Proline arginine-rich end leucine-rich repeat protein-like	LOC100363743	2.05	3.42	**5.41**	1.37	3.36	1.99
1389018_at	100362331	rCG59612-like	LOC100362331	1.81	2.50	**5.21**	0.69	3.40	2.71
1382190_at	266762	MAS-related GPR, member F	Mrgprf	1.63	3.81	**5.15**	2.18	3.52	1.34
1385788_at	287989	Eph receptor B3	Ephb3	0.38	3.55	**5.03**	3.17	**4.65**	1.47
1374942_at	293566	Carboxypeptidase X (M14 family), member 2	Cpxm2	1.01	3.07	**5.03**	2.06	4.02	1.95
1372168_s_at	25641	Insulin-like growth factor binding protein 6	Igfbp6	2.69	**4.58**	**4.94**	1.89	2.25	0.37
1391341_at	303505	Plexin domain containing 1	Plxdc1	0.38	3.51	**4.92**	3.13	**4.53**	1.41
1367700_at	64507	Fibromodulin	Fmod	0.79	1.91	**4.91**	1.12	4.12	3.00
1392510_at	362336	Family with sequence similarity 180, member A	Fam180a	0.56	1.43	**4.71**	0.87	4.16	3.29
1393672_at	289094	Hemicentin 1	Hmcn1	0.20	4.11	**4.57**	3.91	**4.38**	0.46
1373674_at	362429	Microfibrillar associated protein 5	Mfap5	2.21	**4.47**	3.47	2.26	1.25	− 1.01
1376711_at	84588	Claudin 11	Cldn11	2.77	3.75	**4.43**	0.98	1.66	0.68
1384944_at	314423	B-cell CLL/lymphoma 11B (zinc finger protein)	Bcl11b	0.48	4.31	**4.42**	3.83	3.94	0.11
1371700_at	287382	Microfibrillar-associated protein 4	Mfap4	3.29	4.07	**4.41**	0.78	1.12	0.34
1367571_a_at	24483	Insulin-like growth factor 2	Igf2	2.61	3.31	**4.39**	0.70	1.78	1.08
1397360_at	365009	C-type lectin domain family 3, member a	Clec3a	*− 5.90*	*− 6.16*	− 2.72	− 0.26	3.19	3.45
1388973_at	305104	Collagen, type IX, alpha 1	Col9a1	*− 4.53*	*− 5.19*	− 3.20	− 0.66	1.33	1.99
1378873_at	690026	Histone linker H1 domain, spermatid-specific 1	Hils1	− 4.23	*− 5.01*	− 1.62	− 0.79	2.60	3.39
1368711_at	25099	Forkhead box A2	Foxa2	*− 4.43*	*− 4.52*	− 3.06	− 0.08	1.37	1.46
1393943_at	313954	Matrilin 3	Matn3	− 3.27	*− 4.39*	− 3.27	− 1.11	0.00	1.11

^a^For each gene in the pairwise comparison, there were 2 normalized intensity values (one for each group) representing the expression levels. The fold change (FC) value for the gene is calculated by dividing the larger value by the smaller one, then a positive sign is assigned if the gene is upregulated, in other words the gene expression value of the sample (the first group) is greater than the reference (the second group). On the other hand, downregulation of the gene (negative sign) indicates that the gene expression value of the sample is less than the reference.

### Altered canonical pathways

**Figure 1:**
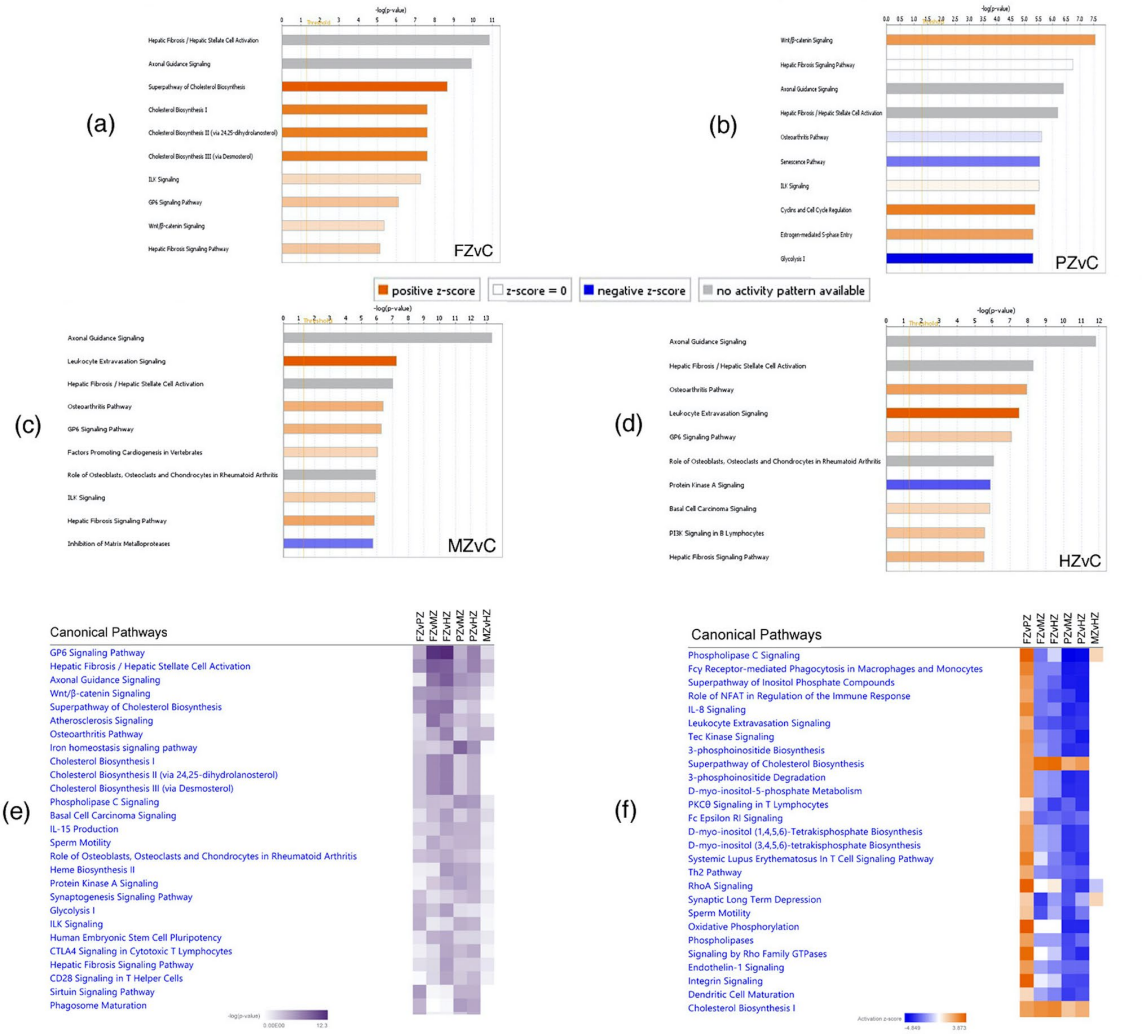
Canonical pathways identified by IPA analysis of the differentially expressed genes. (**a**–**d**) The top ten significant pathways for four pairwise comparisons; each MCC zone compared with FCC (the control). (**e**,**f**) Composite summary showing the identified canonical pathways when comparing MCC zones against each other (six comparisons). The pathways are ranked by the negative log of the p-value of the enrichment score and colored according to the z-score. A positive score indicates a significantly increased function (orange), a negative z-score indicates a significantly decreased function (blue), and an undetermined prediction is shown in gray color. Data were analyzed through the use of IPA (QIAGEN Inc., https://www.qiagenbioinformatics.com/products/ingenuitypathway-analysis)^29^.

**Figure 2:**
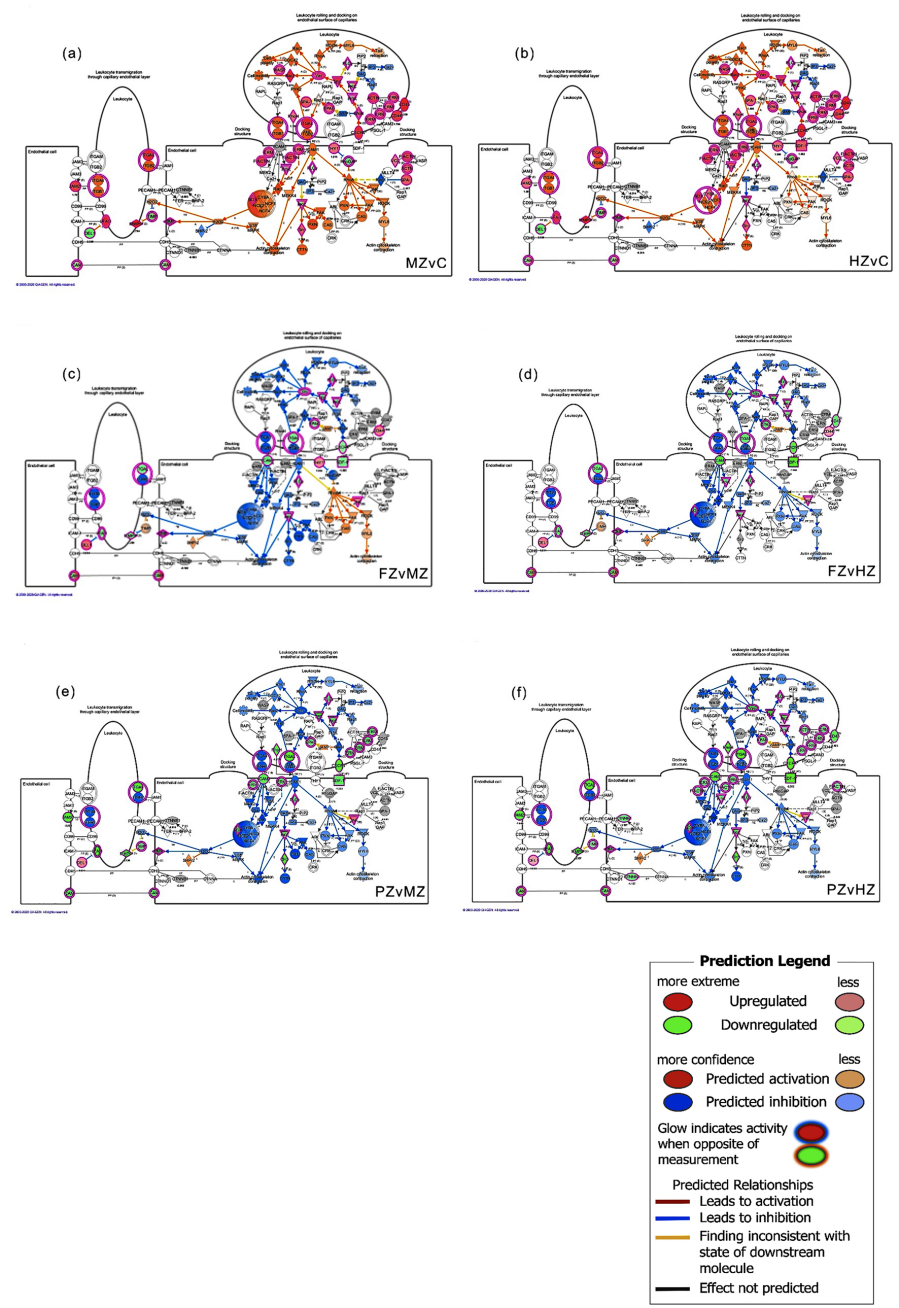
Diagram of Leukocyte extravasation signaling (LES) canonical pathways with overlaid molecular activity prediction as identified by Ingenuity Pathway Analysis (IPA) analysis of the differentially expressed genes in mandibular condylar cartilage zones and femoral condylar cartilage (FCC). The upregulated (red color) and downregulated (green color) genes identified in six comparisons, MZvC (**a**), HZvC (**b**), FZvMZ (**c**), FZvHZ (**d**), PZvMZ (**e**), PZvHZ (**f**) are shown along with predictions on biological function. Cell mobility, cell polarity, tail retraction, and actin cytoskeleton contraction biologic processes were predicted to be activated (orange color) at the deep zones (MZ & HZ) as compared FCC. These processes are predicted to be decreased (blue color) at MCC superficial layer (FZ & PZ) when compared to deeper zones. Data were analyzed through the use of IPA (QIAGEN Inc., https://www.qiagenbioinformatics.com/products/ingenuitypathway-analysis)^29^.

When analyzing genes with FC >  ± 1.4 at threshold p-value < 0.05 for the ten comparisons, ingenuity pathway analysis identified large numbers of altered pathways, ranging from 477 to 548, (the full list of pathways for each comparison is provided in Supplementary Tables 5–14). The top 10 canonical pathways of each MCC zones in comparison to FCC (the control), and the top 27 pathways from the analysis of MCC zones against eachother are shown in Fig. 1a–f. To illustrate the possible changes in biological processes across MCC zones, we used the composite summary tool of IPA, and based on the z-score activation state, there was an overrepresentation of pathways with predicted inhibition at both superficial zones (FZ&PZ) as compared to MCC deeper layers (MZ&HZ) except for two cholesterol biosynthesis pathways (Fig. 1e). Conversely, most of the identified pathways showed predicted activation upon comparing MCC zones against FCC (Supplementary Fig. 12). Proliferative zone (PZ) was found to have more unique differentially expressed pathways, whilst MZvsHZ comparison showed the least significance values and activation scores (Fig. 1e,f, Supplementary Fig. 12). Leukocyte extravasation signaling (LES) pathway was predicted to be activated at all MCC zones in comparison to FCC with increased cell movement of blood cells, interaction of blood cells, invasion of cells, migration of cells, vasculogenesis, cell movement of smooth muscle cells, and migration of muscle cells. Within MCC, cell mobility, cell polarity, tail retraction, and actin cytoskeleton contraction biologic processes were predicted to be inhibitted at superficial zones as compared the deeper ones (Figs. 1c,d,f, 2). On the other hand, Superpathway of Cholesterol Biosynthesis showed positive z-score (predicted activation) in both FZ and PZ comparisons against FCC and deep MCC zones with predicted decrease in 1,25 dihydroxyvitamin D3 biosynthesis (Fig. 1a,f), Conversely, Protein Kinase A (PKA) Signaling was predicted to be inhibited in all MCC zones in comparison with FCC, and remained significantly inhibited at superficial MCC zones as compared the deeper ones (Fig. 1d, Supplementary Fig. 12). PKA is an enzyme regulates other proteins by phosphorylation, thus it is essential for many processes such as metabolic energy, cell survival, cell proliferation, muscle contraction, membrane transport and gene expression. Hepatic Fibrosis/Hepatic Stellate Cell Activation Pathway was also one of the most significant identified pathways in all comparisons, however, its state of activation could not be predicted by IPA (Fig. 1a–d). Similarly, the activation state of other significant pathways e.g. Axonal Guidance Signaling, Hepatic Fibrosis Signaling, and Osteoarthritis Pathway was undetermined.

### Upstream regulators

To provide biological insight to the reported expression changes, we performed upstream regulator analysis (URA). The analysis revealed hundreds of upstream molecules that could explain the differential gene expressions observed in MAA data (Supplementary Table 15). Upstream regulators can be any gene, transcription facor or small molecule that could affect gene expression. The three most activated/inhibited upstream regulators identified by IPA analysis for each of the ten comparisons (total of 35 molecules) are listed in Table 4. These regulators affect various biological processes such as development of body trunk, development of connective tissue cells, development of hematopoietic progenitor cells, metabolism of protein, phosphorylation of protein, inflammation, cellular homeostasis, cell cycle progression, cell–cell contact, activation of connective tissue cells, cell growth and proliferation, transformation, differentiation, movement, migration, as well as cell death and survival. Regulators determined with IPA are sometimes dependent on each other, mechanistic networks are usually constructed to indicate possible signaling mechanisms (Supplementary Fig. 13).

**Table 4:** The three most activated/inhibited upstream regulators identified by IPA analysis.

Pairwise comparison	Predicted activation			Predicted inhibition		
	Upstream regulator	Molecule type	z-score	Upstream regulator	Molecule type	z-score
FZ vs C	TBX2	Transcription regulator	4.596	l-asparaginase	Biologic drug	− 6.211
	TGFB1	Growth factor	4.432	let-7	Microrna	− 5.159
	CSF2	Cytokine	4.361	Calcitriol	Chemical drug	− 4.761
PZ vs C	TBX2	Transcription regulator	4.022	l-asparaginase	Biologic drug	− 4.472
	EGLN	Group	3.917	Calcitriol	Chemical drug	− 3.928
	RICTOR	Other	3.718	Forskolin	Chemical toxicant	− 3.544
MZ vs C	TNF	Cytokine	4.694	l-asparaginase	Biologic drug	− 5.194
	Ige	Complex	4.473	Let-7	Microrna	− 3.874
	Vegf	Group	4.318	CDKN2A	Transcription regulator	− 3.712
HZ vs C	CSF2	Cytokine	4.731	l-asparaginase	Biologic drug	− 4.95
	TNF	Cytokine	4.457	CDKN2A	Transcription regulator	− 3.992
	MITF	Transcription regulator	4.442	ETV6-RUNX1	Fusion gene/product	− 3.852
FZ vs PZ	metribolone	Chemical reagent	6.816	RICTOR	Other	− 6
	TGFB1	Growth factor	6.436	Sirolimus	Chemical drug	− 5.16
	HIF1A	Transcription regulator	5.054	CD 437	Chemical drug	− 4.966
FZ vs MZ	elaidic acid	Chemical—endogenous mammalian	4.088	Cholesterol	Chemical—endogenous mammalian	− 3.807
	SREBF1	Transcription regulator	3.777	VEGFA	Growth factor	− 3.498
	SREBF2	Transcription regulator	3.539	FGFR2	Kinase	− 3.128
FZ vs HZ	elaidic acid	Chemical—endogenous mammalian	4.433	SPI1	Transcription regulator	− 3.709
	SREBF2	Transcription regulator	4.162	l-asparaginase	Biologic drug	− 3.695
	SCAP	Other	3.733	VEGFA	Growth factor	− 3.626
PZ vs MZ	CD 437	Chemical drug	5.248	lipopolysaccharide	Chemical drug	− 5.429
	RICTOR	Other	5.129	Vegf	Group	− 4.937
	ST1926	Chemical drug	4.976	TNF	Cytokine	− 4.809
PZ vs HZ	CD 437	Chemical drug	5.114	IFNG	Cytokine	− 5.039
	ST1926	Chemical drug	5.03	TNF	Cytokine	− 4.86
	RICTOR	Other	4.668	Lipopolysaccharide	Chemical drug	− 4.824
MZ v HZ	CG	Complex	2.385	EGLN	Group	− 2.574
	IL10RA	Transmembrane receptor	2.309	Tamoxifen	Chemical drug	− 2.335
	FOXO3	Transcription regulator	2.236	Filgrastim	Biologic drug	− 2.223

The regulators are ranked by activation Z-score; if ≥ 2 increased activity is predicted, whereas a Z-score ≤  − 2 predicts inhibited activity.

### Validation of microarray data by qRT-PCR

Ten genes from FCC and each MCC zones were selected to validate the MAA data by qRT-PCR. Seven genes out of 10 showed expression patterns in real-time PCR comparable to those of the MAA (Supplementary Figs. 14–23).

## Discussion

Microarray experiments are considered as discovery tools that open up new avenues for research by identifying new gene targets^30^. Although global overview of gene expressions in the studied tissue specimens is made possible, microarrays are basically screening tools that can formulate more targeted research questions and generate well-supported hypotheses rather than proven conclusions^31^. In the present study, LCM, was used to selectively obtain cells from the MCC zones individually. LCM along with MAA were employed to provide new insights into characterizing the four MCC zones. The results support formulating the hypothesis that MCC cells have significantly different patterns of gene expression from those of articular chondrocytes, and more importantly, several genes were found to be expressed variably upon the transition from one zone to another whitin MCC. By demonstrating this spatial (zonal) changes in the gene expression levels, our findings support the hypothesis that cells within the MCC have different phenotypic characteristics. This hypothesis is concordant with a previous finding reported by Hinton et al. who concluded that the prechondroblastic cells (cells of both the fibrous and proliferative zones) have different gene expression profiles from the underlying chondrocytes (cells of both mature and hypertrophic zones) of the MCC^27^.

The difference in gene expression ratios was most obvious between the articular chondrocytes (FZ) and other MCC zones, nevertheless, strong differences were also identified within the other MCC comparisons. In a related study by Fukui et al., zonal differences between the genes of superficial fibroblastic and deep hypertrophic regions of human femoral cartilage were also found to be very pronounced^12^. When comparing adjacent MCC zones such as FZ with PZ, PZ with MZ, and MZ with HZ at ≥ 20 FC level, only three genes were modulated out of the 30 identified genes (Table 3). Indeed, given the strong overlap in the cellular and extracellular composition between the mature and hypertrophic chondrocytes, it is not surprising that significant expression differences are limited to relatively very few genes. On the contrary, all of the 30 differentially regulated genes at ≥ 20 FC level were identified when we compared non-adjacent zones (Table 3). We could neither support or oppose these findings by the literature as there are no similar previous zone-specific studies on the MCC, however, by the analogy with Wang et al. study, where only proliferative and hypertrophic growth plate zones were isolated and then compared, the presence of significant differential gene expression between the two studied zones could be considered supportive to our findings^32^. Likewise, Zhou et al. study identified 804 differentially expressed genes when the articular zone of MCC was compared with the mature zone^33^.

The strongest upregulated relative expression ratio was observed for the Crabp1 gene in FZ as compared to HZ and to the control (7.45- and 7.21-folds respectively) (Tables 2 and 3). Retinoic acids, the active ingredient of vitamin A, play a role in different activities including cellular growth, differentiation and development by binding to specific nuclear receptors, and then regulate gene expression^34,35^. Both vitamin A deficiency and excess lead to skeletal defects; large doses result in growth retardation and premature closure of the growth plate, whereas administration of retinoid antagonists prevents further differentiation of prehypertrophic chondrocytes, indicating the importance of endogenous retinoids for chondrocyte maturation^36^. CRABPs are carrier proteins crucially important for the transport and metabolism of retinoic acid^34^. The amount of the latter substance reaching the nucleus is modulated two cytoplasmic binding proteins CRABP I and II^37^. Overexpression of CRABP I is probably preventing retinoic acid from entering the nucleus by keeping it in the cytoplasm, and by facilitating the acid degradation^38^. On the contrary to our identified bizonal increase of Crabp1in the superficial zones of MCC, a study on rabbit growth plates reported much higher level of Crabp1 transcript in the maturing and hypertrophic chondrocytes than in resting and proliferating chondrocytes^39^. This disagreement could be attributed to the variant cell phenotypes, especially the dividing cell population (PZ) in the MCC as compared with the growth plate.

On the other hand, the most pronounced downregulated gene was Clec3a (− 8.92-fold) in FZ in relation to the femoral cartilage (Table 2). Clec3a gene is a cartilage-derived member of the C-type lectin superfamily. It requires calcium for binding, hence designated as C-type. The protein it encodes is apparently restricted to cartilage and involved in many biologic functions as it promotes cell adhesion to laminin-332 and fibronectin. While this protein has been found in nucleus pulposus, nasal cartilage and in articular cartilage, the distribution of mRNA of Clec3a in the developing rib was related to the upper hypertrophic and proliferating chondrocyte zones, suggesting a role in organizing the ECM and probably in regulating the epiphysis remodeling^40^. According to our MAA data, Clec3a was the most downregulated gene at FZ; a similar expression pattern was demonstrated by Grogan et al., who studied the zonal expression patterns of genes in the FCC of human and bovine. They found a significant downregulation of Clec3a gene in the superficial zone compared to the middle zone^41^. It is worth mentioning that some of the differentially expressed genes identified in the present study were not reported previously. This is in concordance with Hinton et al. study where novel unsuspected genes were differentially expressed in the perichondrium of the MCC^27,42^. Furthermore, the identification of relatively large number of unknown genes and expressed sequence tags may indicate that novel molecular pathways are not yet identified^43^. Intriguingly, 25.3% of the differentially expressed genes in at least one of the ten pairwise comparisons conducted at ≥ 20-fold cut-off value were unknown genes (Supplementary Table  2); thus the current study implies that several yet-to-be identified pathways may play a significant role in MCC.

In osteoarthritis, proteolytic enzymes such as matrix metalloprotease and aggrecanases degrade cartilage extracellular matrix components. This is accompanied with the expression of hypertrophic chondrocytes markers e.g. type 10 collagen (COL10A1), vascularization, and focal calcification. These features are similar to the normal endochondral ossification process that takes place in the growth plate^44^, where proliferating chondrocytes secrete Chondromodulin-I, Tenomodulin, and Sox to inhibit angiogenesis, while hypertrophic chondrocytes promote angiogenesis through hypoxia-inducible factor 1 (HIF) and vascular endothelial growth factor (VEGF) signaling to and recruit blood vessel invasion^45^. Inflammation and angiogenesis are closely correlated; angiogenesis may enable leukocyte extravasation into tissues by increasing the total endothelial surface, and several cytokines, chemokines, CAMs (cell adhesion molecules), and growth factors can also modulate neovascularization^46^. We predicted Leukocyte extravasation signaling (LES) pathway to be activated in MCC (Fig. 1c,d,f), in particular at deeper zones where chondrocytes hypertrophy very rapidly^42^ (Fig. 2a,b). Leukocyte recruitment into tissue across the endothelium requires four steps: rolling, tethering, firm adhesion, and diapedesis^47^, and involves the participation of different adhesion receptors such as selectins, integrins and immunoglobulin superfamilies^48^. In our IPA, matrix metalloproteinases (MMP3, MMP9, MMP10, MMP12, MMP14, and MMP28), chemokinases (CXCL12,CXCR4), and claudins (CLDN11,CLDN22,CLDN5) were differentially expressed in relation to LES canonical pathway (Supplementary Tables 5–14). MMPs are enzymes which can degrade collagen, proteoglycans, and other extracellular matrix components, simultaneously. These enzymes are tightly regulated by several growth factors, cytokines, specific tissue inhibitors of MMPs (TIMPs)^49^. The abundance of MMP9, MMP12 and TIMP1 in MZ & HZ of the MCC, alongwith the substantial downregulation of MMP3, TIMP3 and TIMP4 shown in our results further affirm the importance of balancing the expression of MMPs to TIMPs in cartilage microenvironment to maintain its integrity^49^. In corroboration of the crucial role of chemokines in leukocytes recruitment, we found CXCL12, CXCR4 to be substantially expressed in the deep layers of MCC. Chemokines are chemoattractant cytokines that stimulate cell movement and migration signaling events, in particular leuckocyte trafficking, they also induce many other biologic processes such as cell proliferation, survival, development, and angiogenesis under both physiological and pathological conditions^50,51^. In addition to matrix metalloproteinases and chemokines, our results demonstrated modulation of three members of cluadins family. Studies have shown that claudins, which are integral membrane proteins and tight junction proteins, may be involved in cell adhesion^52^. Claudin 11 (CLDN11), a major component of central nervous system (CNS) myelin, was abundant prenatally in developing meninges, mesoderm, and adjacent to cartilage, indicating its major role in growth and differentiation of not only oligodendrocytes but also other cells outside CNS^53^.

Lipids such as phospholipids, cholesterol and fatty acids in cartilage are important as source of energy for cells. They are also an essential constituent of cellular membranes, and play a role as signalling molecules^54,55^. High cholesterol levels are associated with osteoarthritis, whereas cholesterol synthesis inhibition reported to be associated with skeletal dysplasias; confirming the important role of cholesterol biosynthesis in chondrogenesis^56^. Genes-encoding proteins necessary for cholesterol biosynthesis, such as acetyl-coenzyme A acetyltransferase 1 (ACAT1), cytochrome P450 oxidase, family 51, sub-family A, polypeptide 1 (CYP51A1), 3-hydroxy-3-methylglutaryl-coenzyme A synthase 1 (HMGCS1) or 7-dehydrocholesterol reductase (DHCR7), have been detected to be highly expressed in the superficial zones of MCC (Supplementary Table 1) where Superpathway of Cholesterol Biosynthesis is also predicted to be activated according to our results (Fig. 1a,e,f),. Previously reported data has shown that cholesterol and lipid biosynthesis are crucial for regulation of differentiation, proliferation and apoptosis of undifferentiated mesenchymal cells in the growth plate, probably via regulating many other signaling pathways, such as Wnt signaling and Hedgehog (Hh) signaling^56,57^. Upstream regulator analysis of our microarray data identified RICTOR (rapamycin-insensitive companion of mTOR), SREBF1, SREBF2 (SREBPs are sterol regulatory element-binding proteins), and SCAP (SREBP cleavage-activating protein), which have been implicated in the process of cholesterol synthesis, among the most activated upstream regulators in the superficial FZ & PZ of MCC (Table 4 and Supplementary Fig. 13). The mammalian target of rapamycin (mTOR) is a serine/threonine protein kinase that regulates the phosphorylation of many proteins, and has two functional complexes; mTORC1 and mTORC2. RICTOR, which is a subunit of mTORC2, regulates cell metabolism, growth, proliferation and survival in response to growth factors and hormonal signals^54,58^. In addition to protein synthesis, mTOR is also a critical regulator of lipid biosynthesis through SREBF1/SREBP1 but little is known about mTOR lipid-induced responses in chondrocytes. SREBPs and SCAP regulate intracellular cholesterol biosynthesis, when cholesterol levels are low, SCAP/SREBP complex allows proteases to cleave SREBP and then to traffic to the nucleus where target genes for the biosynthesis of cholesterol are activated. Conversely, when intracellular levels are high, cholesterol biosynthesis is prevented by tethering the SREBP/SCAP complex to the endoplasmic reticulum membrane. Studies showed that Hedgehog signaling and intracellular cholesterol synthesis regulate each other. Activation of this signaling pathway, which regulates SCAP expression, induces cholesterol accumulation, which is crucial for chondrocytes proliferation and differentiation^56,57^. The predicted activation of Superpathway of Cholesterol Biosynthesis and of RICTOR, SREBF1, SREBF2 and SCAP upstream regulators at FZ and PZ of MCC (Fig. 1, Table 4, Supplementary Fig. 13), is consistent with that the undifferentiated cells of these superficial zones have high metabolism and require high levels of cholesterol and lipids, whereas the differentiated or nearly differentiated cells of the deeper zones (MZ &HZ) exhibited comparatively predicted inhibition of such regulators in our bioinformatic analysis.

It is evident that functional crosstalks exist between the signaling pathways involved in endochondral ossification process. Interestingly, studies showed that Hh signaling crosstalks with the Notch signaling, fibroblast growth factor (FGF) pathway, Wingless-related integration site (Wnt) signaling, bone morphogenetic protein (BMP) signaling, and mTOR signaling pathways^44^. Likewise, Wnt pathway may interact with BMP, Hh, FGF and TGF-β (transforming growth factor) signaling pathways^59^. Another intriguing feedback loop between PTHrP (Parathyroid-hormone-related protein) and Ihh (Indian hedgehog) signaling pathways was found to be involved in the homeostasis of articular cartilage and growth plate cells^44^. Furthermore, Hedgehog signaling can regulate cholesterol homeostatic genes; indicating a feedback loop in chondrocyte differentiation^56,57^. Unraveling the underlying mechanisms of these feedback loops and crosstalks will further provide important insights and enable better understanding of such interactions which take place in cartilaginous tissues. While numerous underlying pathways still remain unknown, IPA of zone-specific microarray data generated an abundance of data with large number of differentially expressed genes, and identified lists of activated/inhibited different signaling pathways and upstream regulators (Supplementary Tables 1–15). All of these cannot be introduced and discussed in this study but one cautionary note when interpreting bioinformatic data is to categorize the identified molecules and/or genes as either suppressors or promoters with caution. Rather than this binary assignment, it is strongly recommended to evaluate it as highly specialized and complicated balance of several bioactive molecules that is needed to maintain tissue homeostasis.

Since numerous properties are shared, rat MCC was chosen as a model of normal developmental processes taking place in the human MCC. In the present study, we selected the age of 5 weeks not only because MCC articulation function is already present in a more mature state, but also the maximum growth spurt for rats occurs at day 31.5. Accordingly, this age will allow studying and detecting genes expression profiles at a larger and broader scale in relation to both articulation and growth functions. Investigating normal conditions at different ages can be considered as baseline studies for future disease-related studies. Studying older age groups is also valuable, especially for evaluating osteoarthritic changes and cartilage degeneration. Gender is another important factor, literature showed that 80% of individuals seeking treatment for TMJ disorders are females of childbearing age. Such a high prevalence suggests a role for female hormones, particularly estrogen, in the disease process. In fact, this is the reason behind not selecting female rats as an animal model. Nevertheless, we consider this experiment as a baseline for future zone-specific studies of the mandibular condylar cartilage at which both male and female genders at different age groups can be studied and compared against each other.

One of the drawbacks of the MAA experiments is the incomplete relevance between the transcripts level determined and the corresponding proteins level. The fact that the differential expressions in mRNA do not necessarily reflect similar changes in proteins could be attributed to that the MAA technology is not related to posttranslational changes and posttranscriptional regulations^60,61^. Another limitation in this study was conducting the MAA experiment with no replicates. Although replication is needed to improve the data quality, the appropriate number of replicates is largely dependent on the research question to be answered. For instance, more replicates are required to confidently identify novel genes^62^, conversely, if the purpose of the study is to formulate a well-ground hypothesis, the issue of sample replicates is not very critical, specially if the MAA is combined with other more sensitive molecular analysis for validation such as qPCR. The limited availability of sample material in our study (very small MCC zones in size) and the relatively high cost of microarray chips and LCM kits have limited the number of biological or technical replicates. While noting that there are no firm standards on the number of replicates required in a microarray chip experiment, Bryant et al. found that the variability attributable to technical and biological variation in a typical in vitro microarray experiment in humans is low, and markedly less than the effect on gene expression of stimulation (MCC zonal architecture in our case)^63^. Additionally, MAA experiments designs that allow multiple independent estimates of treatment effects may allow reduced replication, or even no replication as stated by Maindonald et al.^64^. Such design was applied in our experiment when the four MCC zones were compared against each other^64^. For example, there are two estimates of the comparison between FZ and PZ: one obtained directly by comparing the two zones, and the other estimate is obtained by subtracting the FZ versus C effect from the PZ versus C effect. At the end, the results will provide an overview to allow one to claim that hypotheses can be formulated and prioritized for later work. However, for all the limitations, the current study revealed several new aspects in relation to MCC cell phenotypes, which may offer some clues to research process in this area and contribute to the future therapeutic approaches for MCC diseases and conditions.

In summary, by using a rat genome expression array with more than 31,000 probe sets, a comprehensive evaluation of genome-wide expressions was possible using LCM and MAA technologies, and robust gene expression differences were revealed, supporting the hypothesis that differential gene expression exists between articular chondrocytes of the FCC and MCC cells on the one hand, and different gene profiles exist among the four zones of the MCC on the other hand.

The current study also demonstrated that the MCC zones clearly exhibited differences in the activation/inhibition status of many canonical pathways which appear to be largely dependent on spatial (regional) expression of multiple factors that connect different signaling pathways leading to cartilage/chondrocyte development, maturation and homeostasis. Our results can undoubtedly be used in the future studies for exploring gene–gene interactions and signaling cascades which is crucial for the discovery of new therapeutic strategies for this intriguing cartilaginous tissue.

## Supplementary Information

Supplementary Information 1.

Supplementary Information 2.

Supplementary Information 3.

Supplementary Information 4.

Supplementary Information 5.

Supplementary Information 6.

Supplementary Information 7.

## Data Availability

The authors confrm that the data supporting the fndings of this study are available within the article and its supporting materials.
