# Supplementary material for: Implications of zonal architecture on differential gene expression profiling and altered pathway expressions in mandibular condylar cartilage
Source: Sci Rep. 2021 Aug 19;11:16915. doi: 10.1038/s41598-021-96071-7 (PMC8376865; doi:10.1038/s41598-021-96071-7)
Supplement: Supplementary file 1 — Supplementary Information 1. [file 41598_2021_96071_MOESM1_ESM.docx]

**Implications of zonal architecture on differential gene expression profiling and altered pathway expressions in mandibular condylar cartilage**

**Aisha M. Basudan^1,*^, Mohammad Azhar Aziz^2^ & Yanqi Yang^3^**

^1^ Division of Orthodontics, Dental Services Department, King Abdulaziz Medical City (KAMC) / King Abdullah International Medical Research Center (KAIMRC) / King Saud bin Abdulaziz University for Health Sciences (KSAU-HS), Ministry of National Guard-Health Affairs, Riyadh, 11426, Saudi Arabia.

^2^ King Abdullah International Medical Research Center (KAIMRC) / King Saud bin Abdulaziz University for Health Sciences (KSAU-HS), Colorectal Cancer Research Program, Ministry of National Guard-Health Affairs, Riyadh, 11426, Saudi Arabia.

^3^ Division of Paediatric Dentistry and Orthodontics, Faculty of Dentistry, The University of Hong Kong, 34 Hospital Road, Hong Kong SAR, China.

* Corresponding author A.M.B. (email: aisha_basudan@yahoo.com)


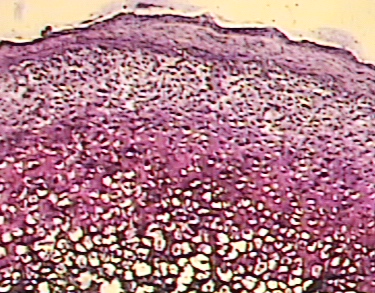


**FZ**

**PZ**

**MZ**

**HZ**

(a)


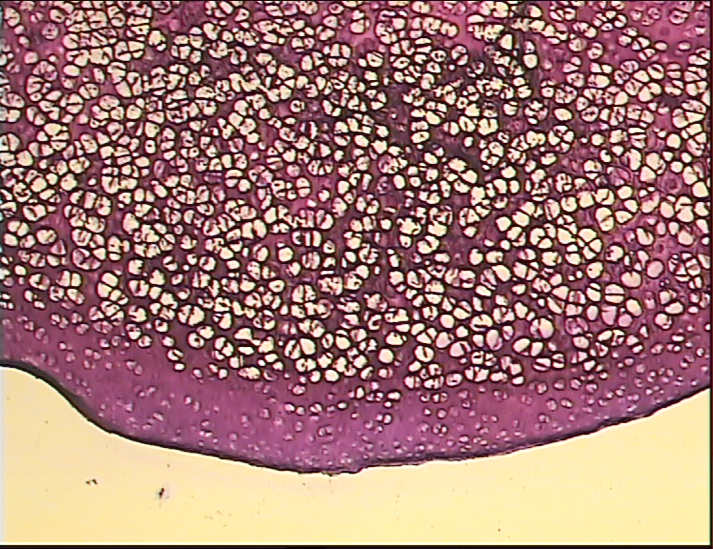


**SZ**

**MZ**

**DZ**

(b)

**Supplementary Figure 1** Zonal architecture of cartilages in 5-week-old rat. (a) Mandibular condylar cartilage (MCC) is divided into: FZ, fibrous zone; PZ, proliferative zone; MZ, mature zone; and HZ, hypertrophic zone. (b) Femoral condylar cartilage (FCC) is also histologically divided into: SZ, superficial zone; MZ, middle zone; and DZ, deep zone. The chondrocytes of the MZ & DZ were microdissected.

**Supplementary Figure 1** Zonal architecture of cartilages in 5-week-old rat. (a) Mandibular condylar cartilage (MCC) is divided into: FZ, fibrous zone; PZ, proliferative zone; MZ, mature zone; and HZ, hypertrophic zone. (b) Femoral condylar cartilage (FCC) is also histologically divided into: SZ, superficial zone; MZ, middle zone; and DZ, deep zone.
