# Supplementary material for: Implications of zonal architecture on differential gene expression profiling and altered pathway expressions in mandibular condylar cartilage
Source: Sci Rep. 2021 Aug 19;11:16915. doi: 10.1038/s41598-021-96071-7 (PMC8376865; doi:10.1038/s41598-021-96071-7)
Supplement: Supplementary file 2 — Supplementary Information 2. [file 41598_2021_96071_MOESM2_ESM.docx]

**Implications of zonal architecture on differential gene expression profiling and altered pathway expressions in mandibular condylar cartilage**

**Aisha M. Basudan^1,*^, Mohammad Azhar Aziz^2^ & Yanqi Yang^3^**

^1^ Division of Orthodontics, Dental Services Department, King Abdulaziz Medical City (KAMC) / King Abdullah International Medical Research Center (KAIMRC) / King Saud bin Abdulaziz University for Health Sciences (KSAU-HS), Ministry of National Guard-Health Affairs, Riyadh, 11426, Saudi Arabia.

^2^ King Abdullah International Medical Research Center (KAIMRC) / King Saud bin Abdulaziz University for Health Sciences (KSAU-HS), Colorectal Cancer Research Program, Ministry of National Guard-Health Affairs, Riyadh, 11426, Saudi Arabia.

^3^ Division of Paediatric Dentistry and Orthodontics, Faculty of Dentistry, The University of Hong Kong, 34 Hospital Road, Hong Kong SAR, China.

* Corresponding author A.M.B. (email: aisha_basudan@yahoo.com)


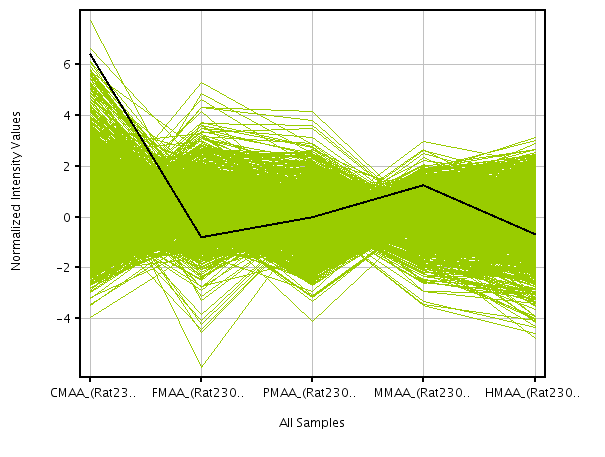

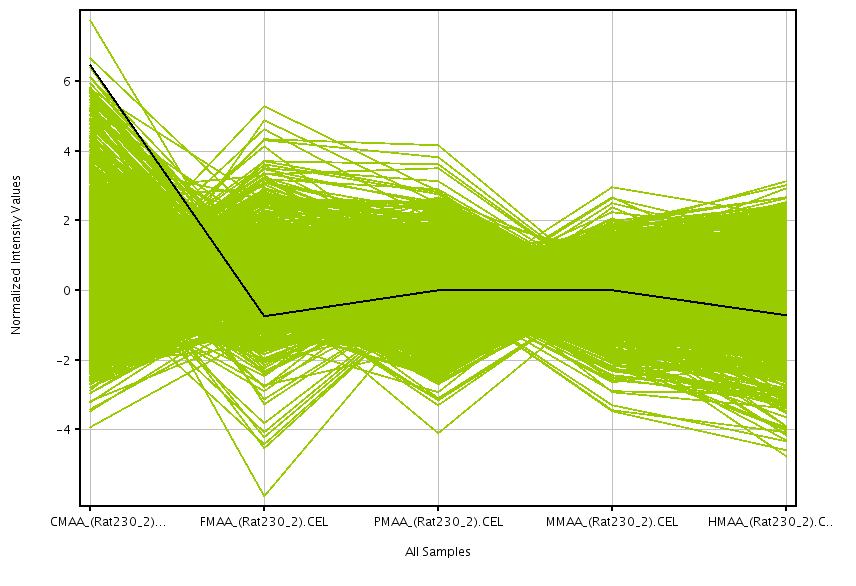


**Mfi2**

**Mia**

**Normalized Intensity Values**


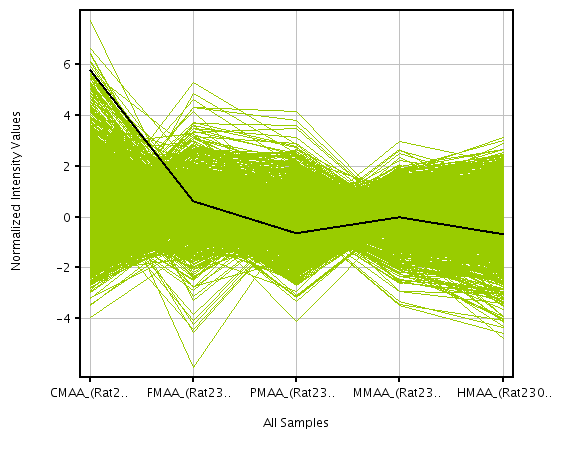

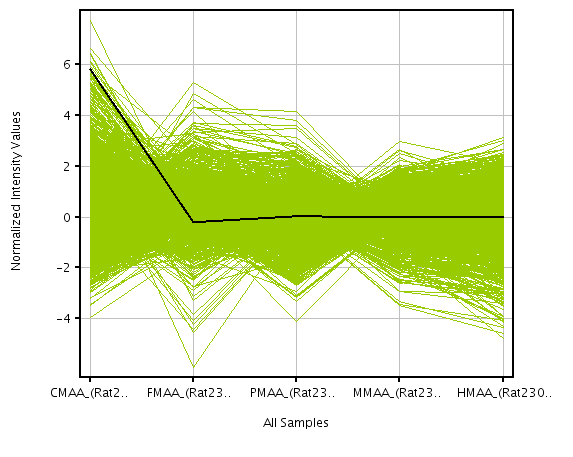

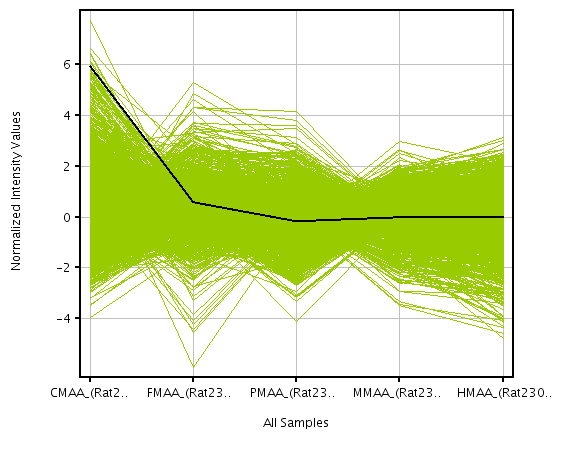

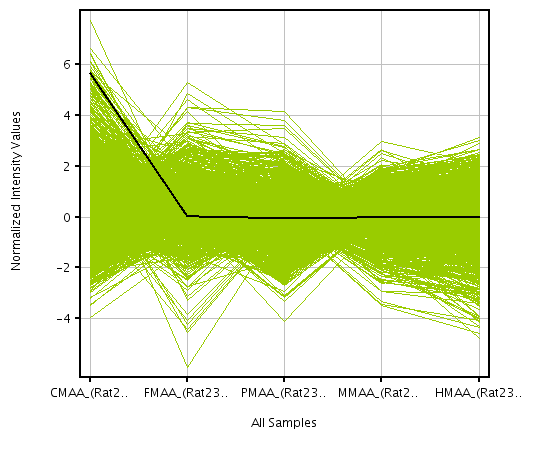

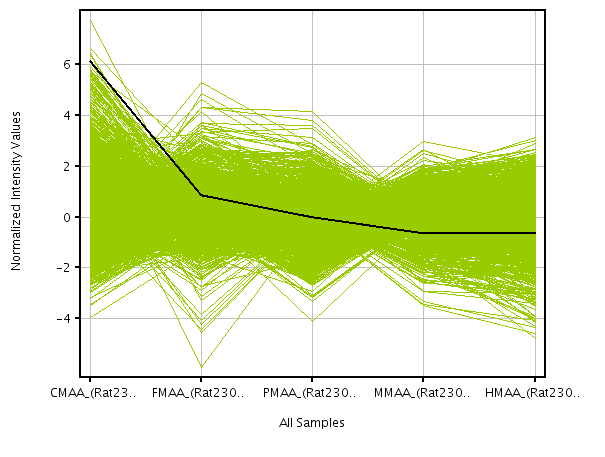


**RGD1566401**

**Gdf10**

**Hoxc9**

**Chad**


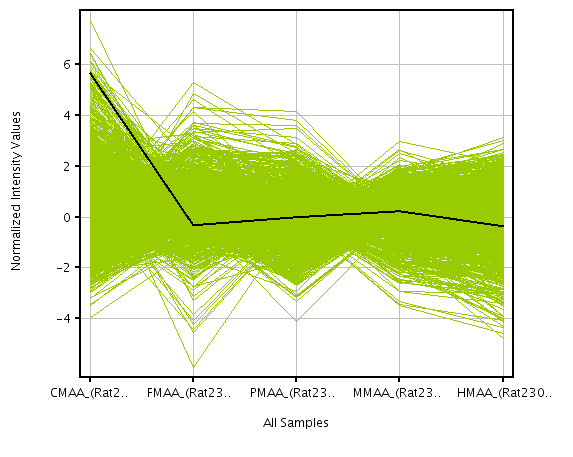

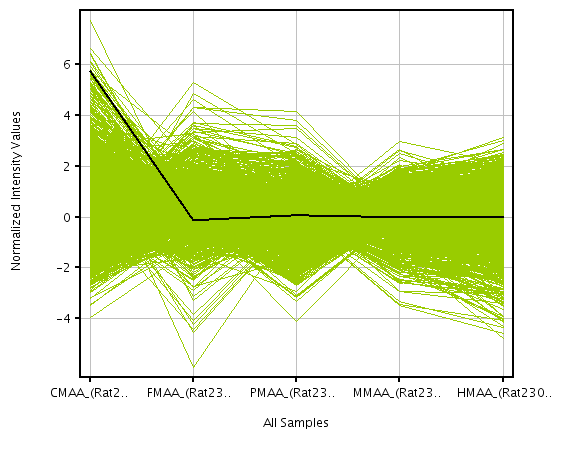


**Hoxc10**

**A1i3/Mug1**

**Ptgds**


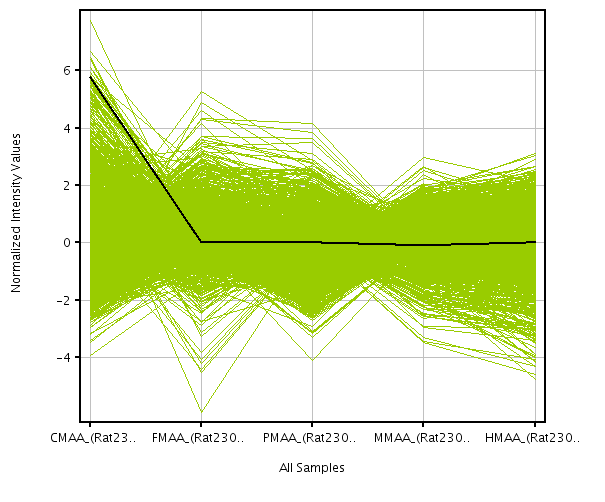


**Hoxc9**

**Normalized Intensity Values**

**Normalized Intensity Values**

**Supplementary Figure 2** Individual profile plots for the top ten annotated genes with the greatest absolute expression values detected in group C, which was dissected from the FCC tissue (black line) in relation to the overall expression profile plot for FCC and MCC zones (green background).


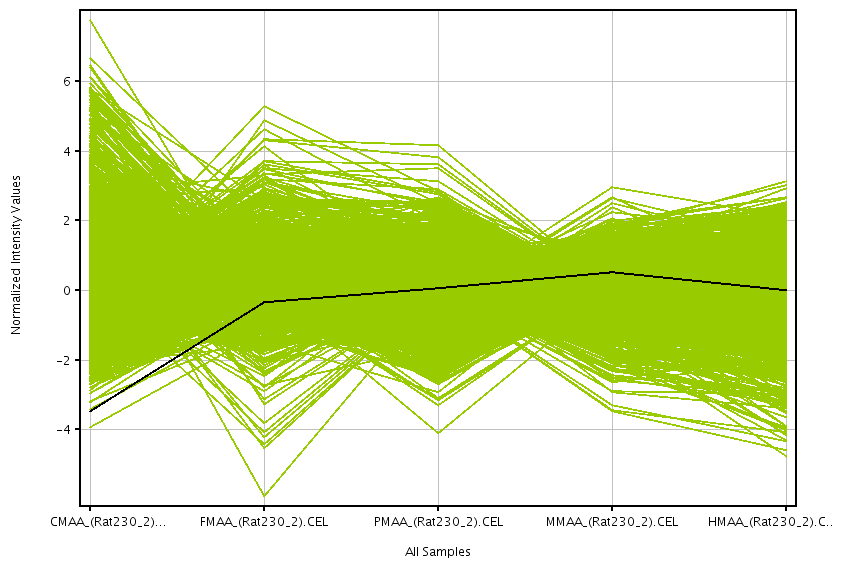

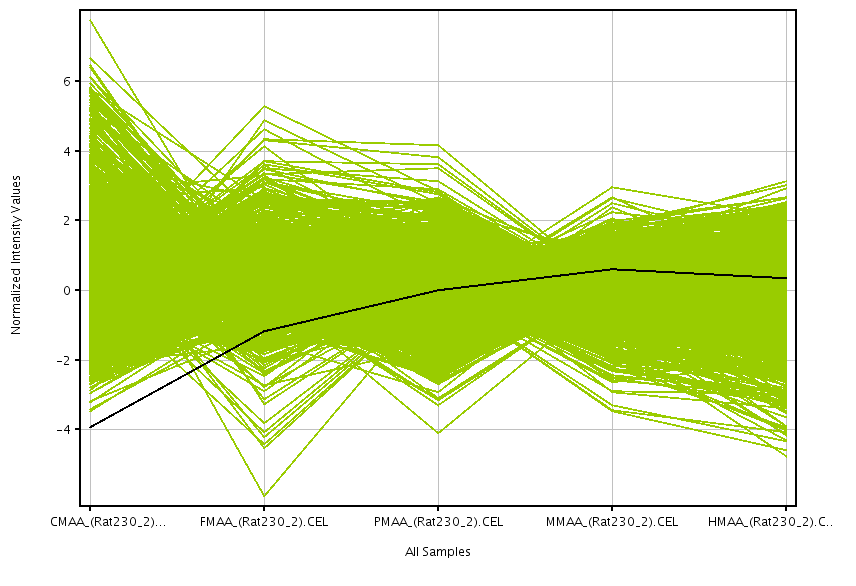


**Normalized Intensity Values**

**Dusp27**

**LOC 685277**


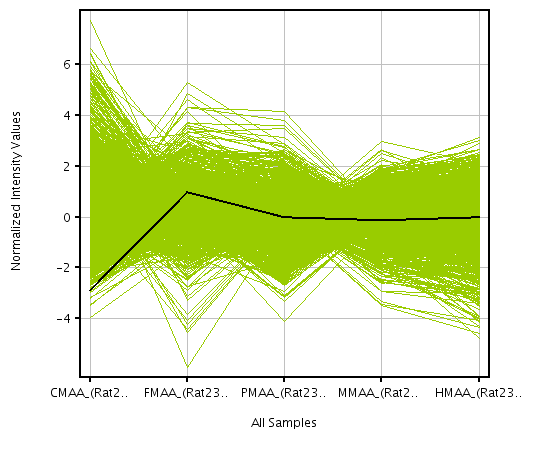

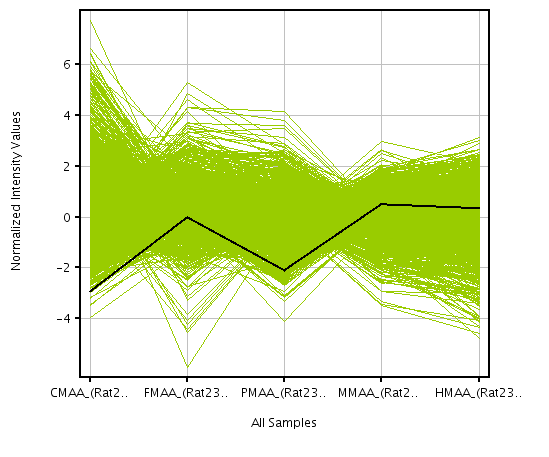

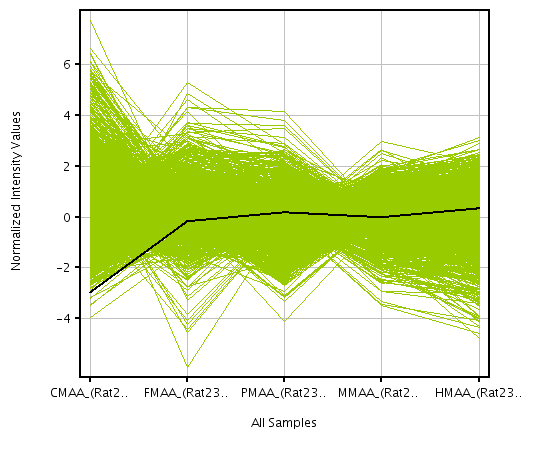

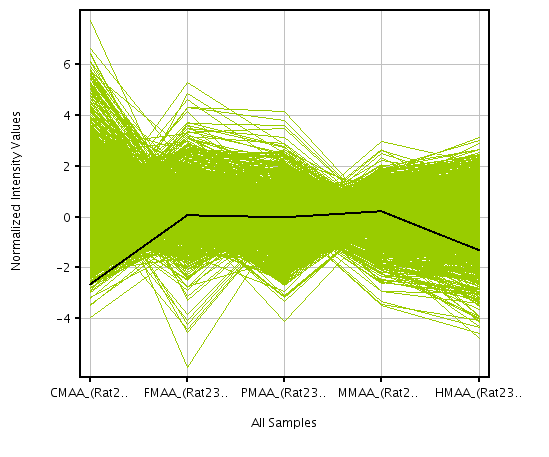

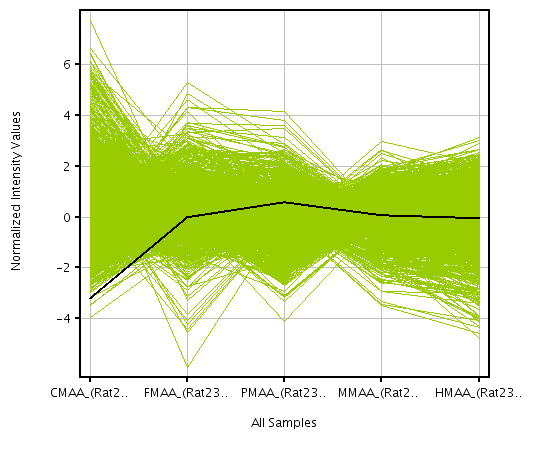

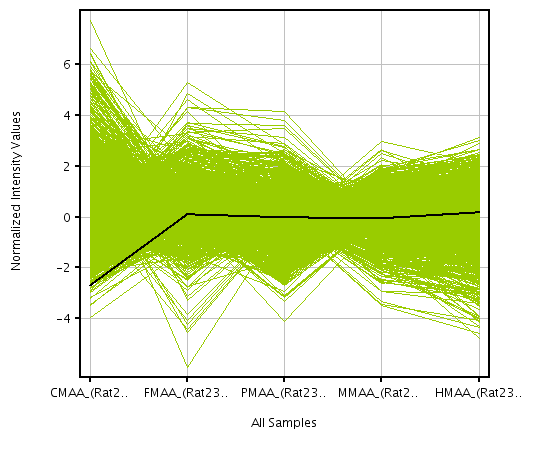

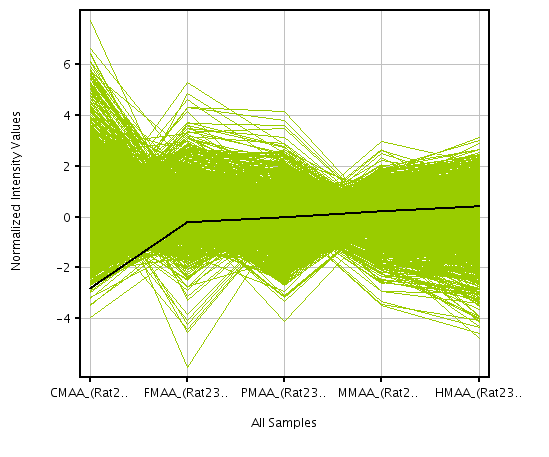

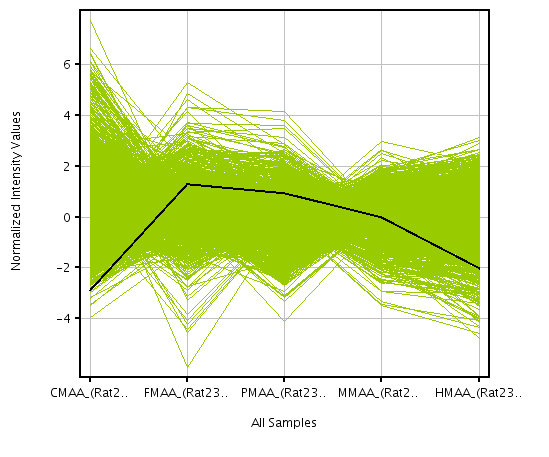


**Normalized Intensity Values**

**Normalized Intensity Values**

**Supplementary Figure 3** Individual profile plots for the top ten annotated genes with the least absolute expression values detected in group C, which was dissected from the FCC tissue (black line) in relation to the overall expression profile plot for FCC and MCC zones (green background).

**Robo2**

**Lox**

**Serpinf1**

**Dlx1**

**Rasal2**

**Fam25a**

**Slfn3**

**Tes**


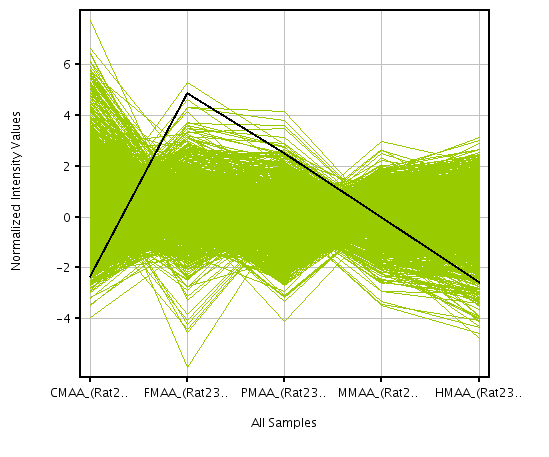

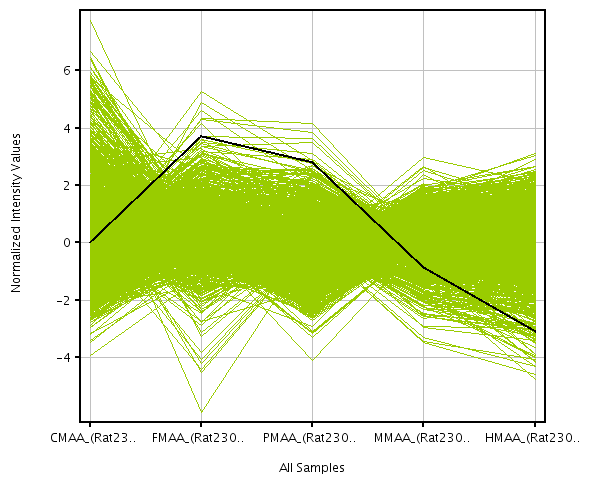

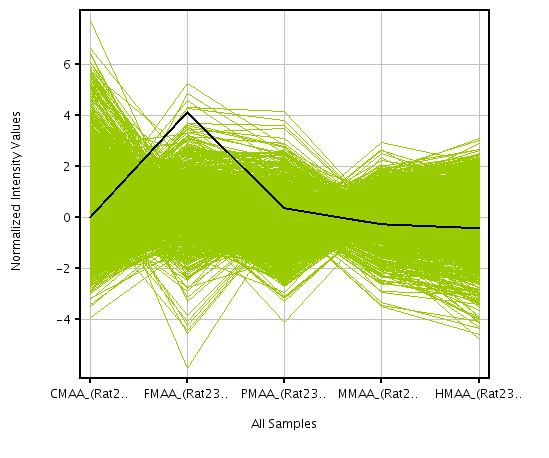

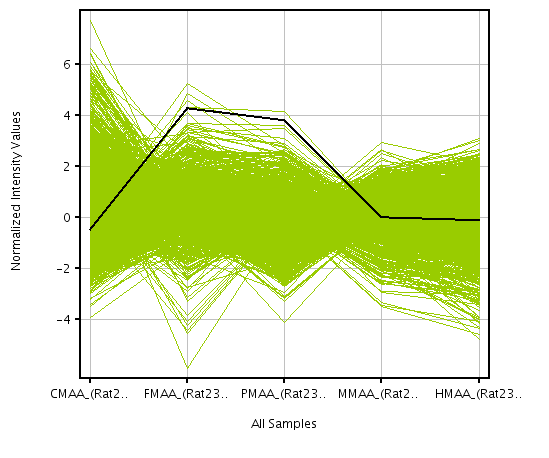

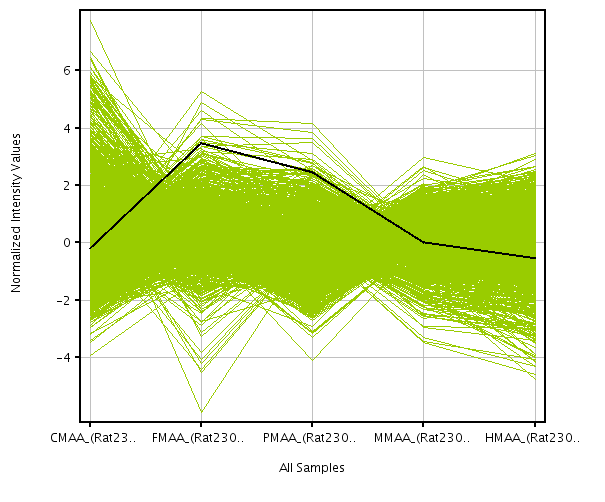

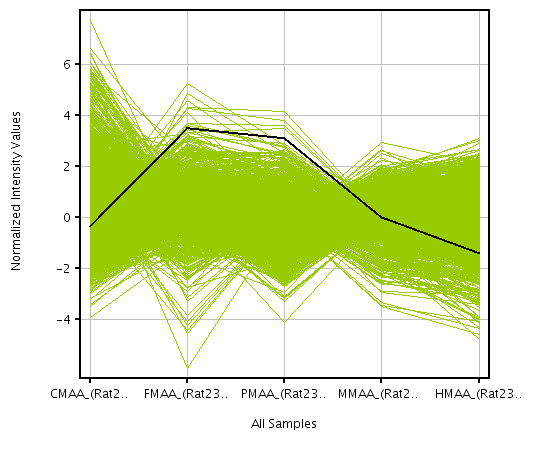

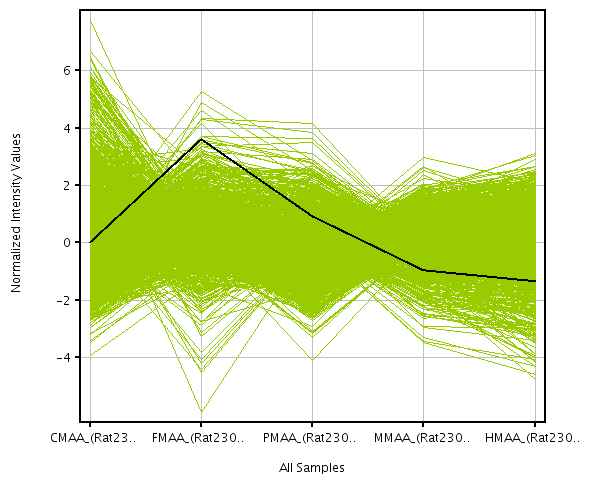

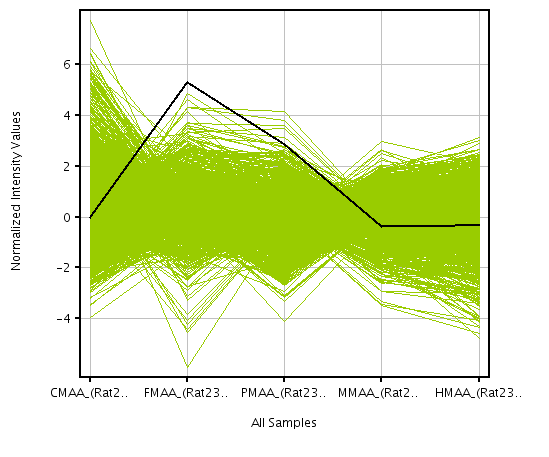

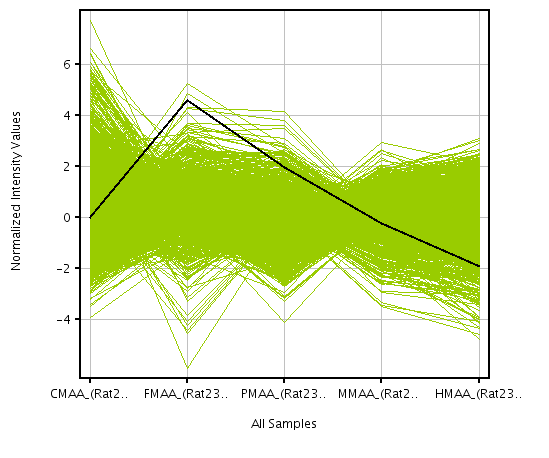

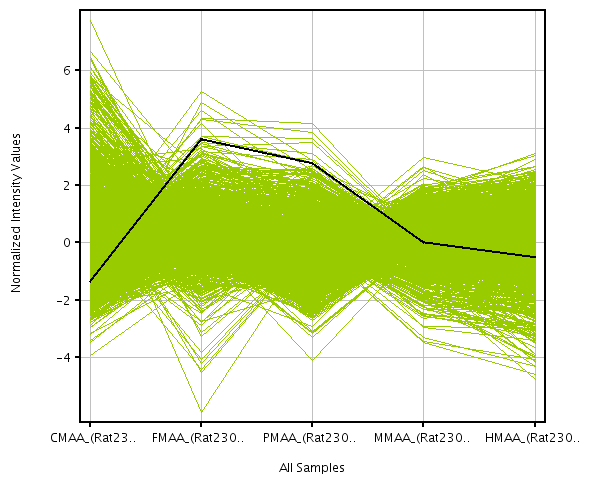


**Normalized Intensity Values**

**Normalized Intensity Values**

**Normalized Intensity Values**

**Tnmd**

**Crabp1**

**Dpt**

**Bcl11b**

**Igfbp6**

**Dpt**

**Plxdc1**

**Bcl11b**

**Igfbp6**

**Plxdc1**

**Supplementary Figure 4** Individual profile plots for the top ten annotated genes with the greatest absolute expression values detected in group F, which was dissected from zone F of the MCC tissue (black line) in relation to the overall expression profile plot for FCC and MCC zones (green background).


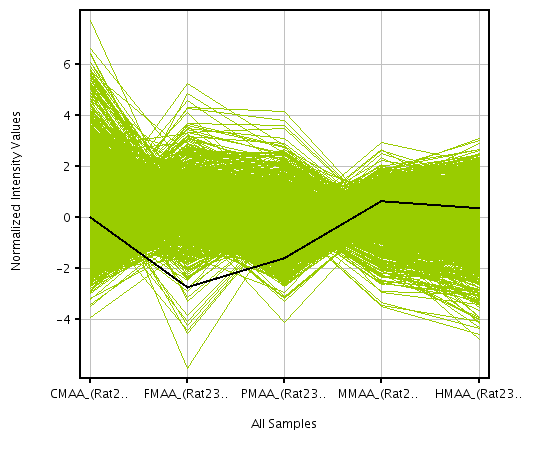

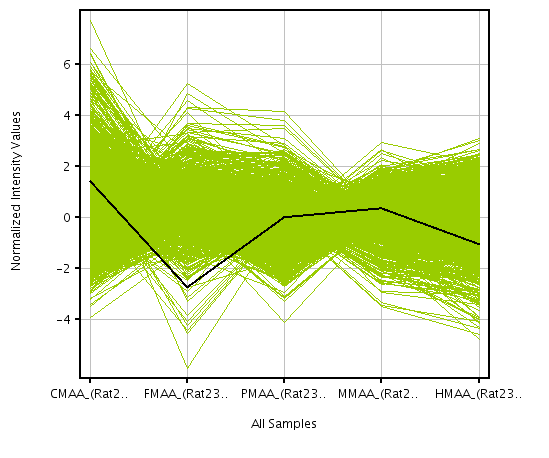

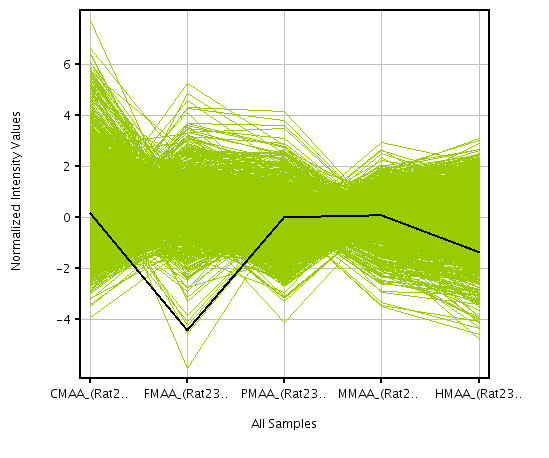

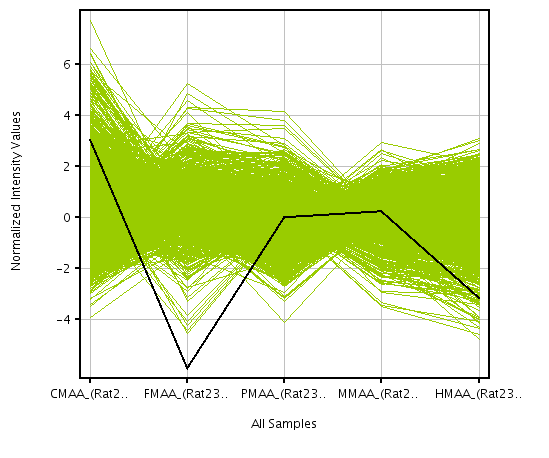

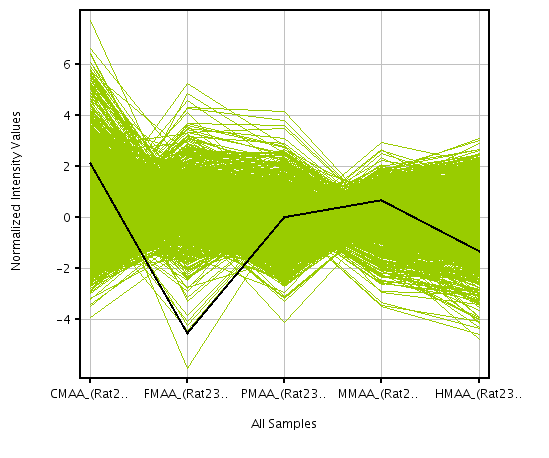

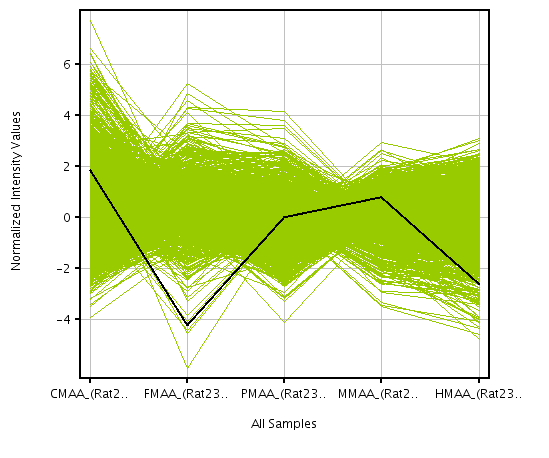

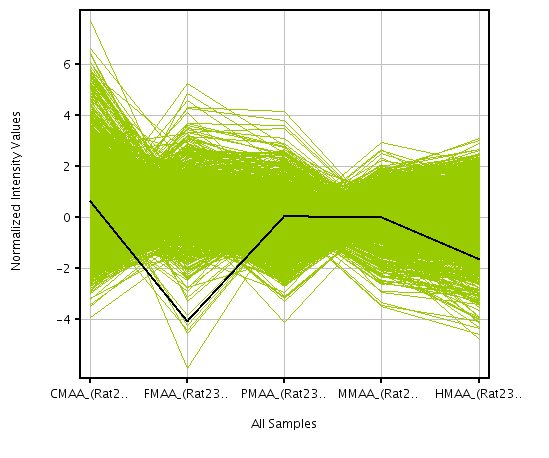

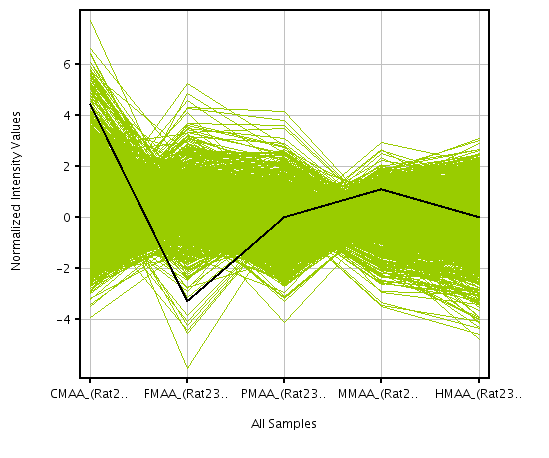

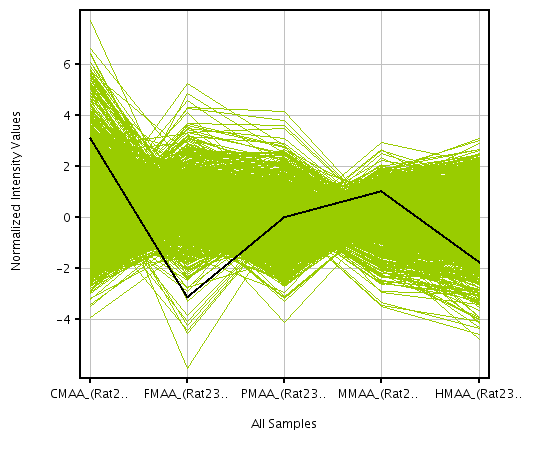

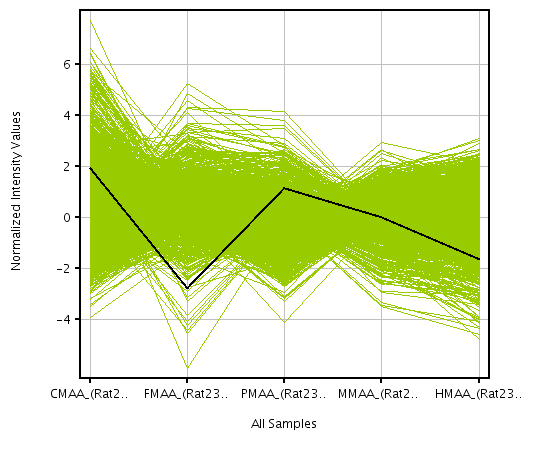


**Normalized Intensity Values**

**Normalized Intensity Values**

**Normalized Intensity Values**

**Supplementary Figure 5** Individual profile plots for the top ten annotated genes with the least absolute expression values detected in group F, which was dissected from zone F of the MCC tissue (black line) in relation to the overall expression profile plot for FCC and MCC zones (green background).

**Clec3a**

**Col9a1**

**Foxa2**

**Hils1**

**Col9a3**

**Matn3**

**Cmtm5**

**Mcoln2**

**Col9a2**

**Col10a1**


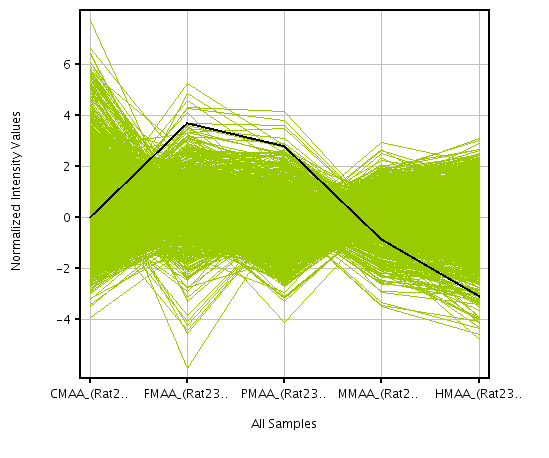

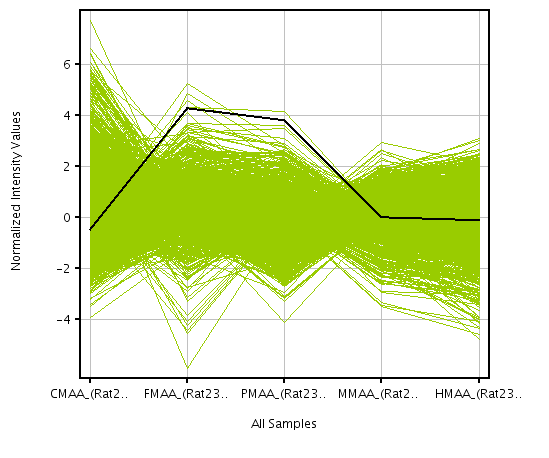

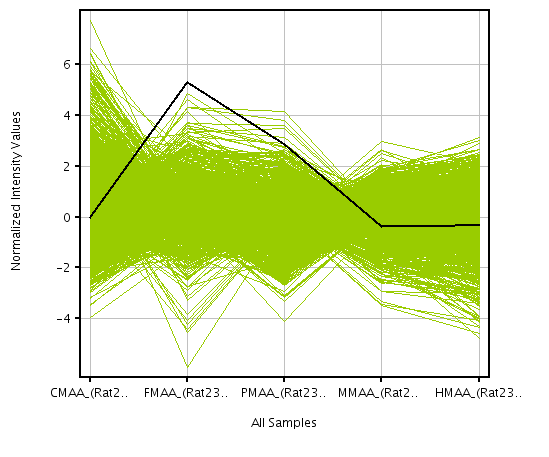

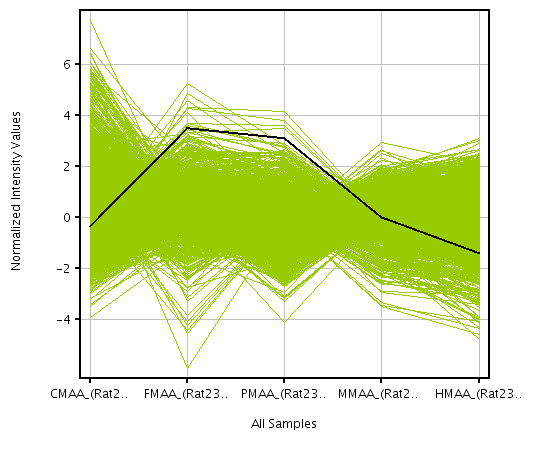

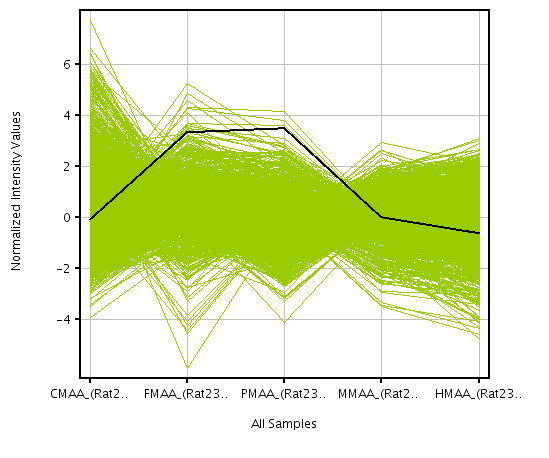

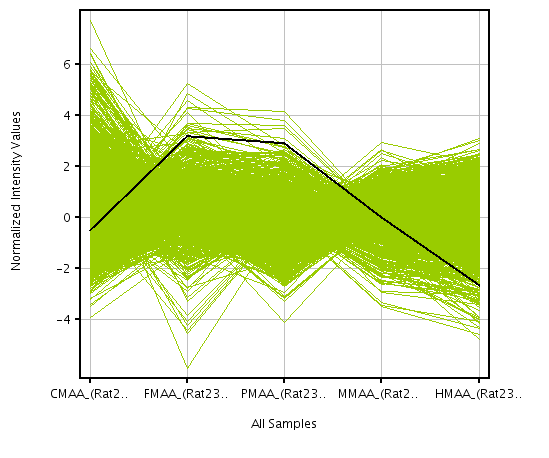

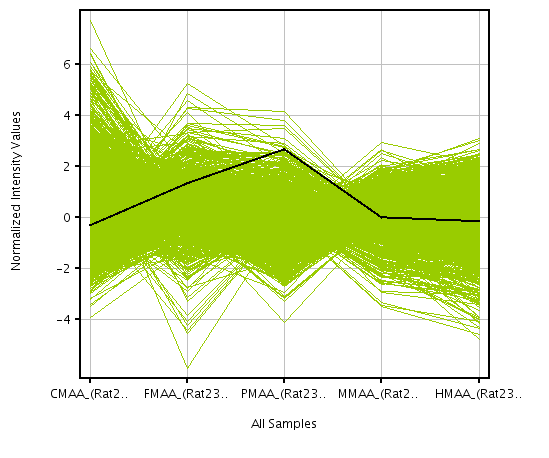

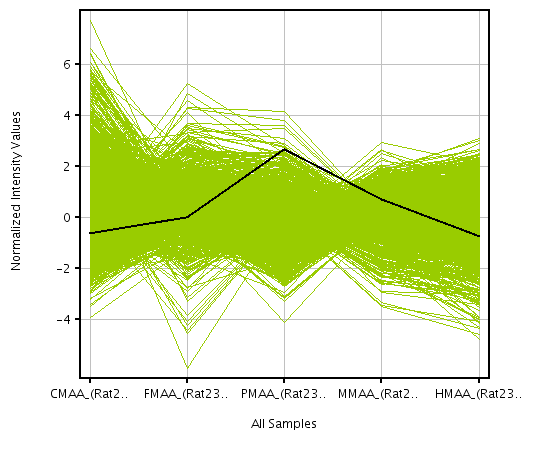

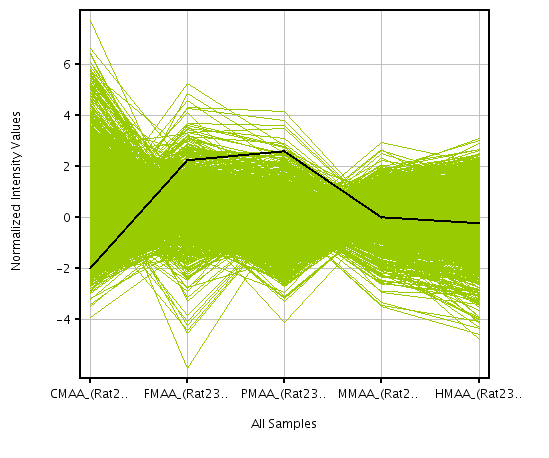

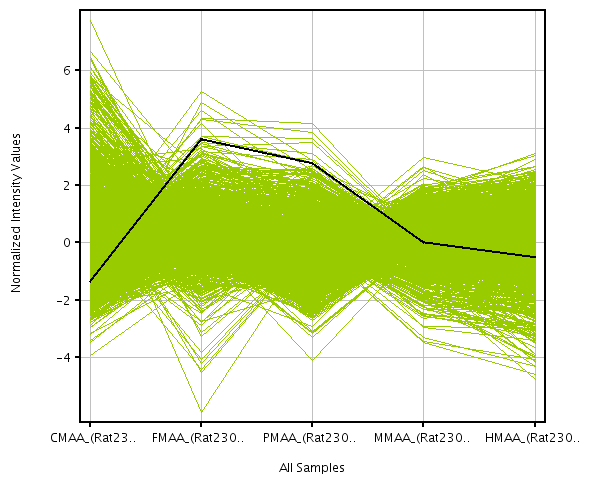


**Normalized Intensity Values**

**Normalized Intensity Values**

**Normalized Intensity Values**

**Bcl11b**

**Tnmd**

**Plxdc1**

**Dpt**

**P4ha3**

**Aspn**

**Hs3st6**

**LOC688502**

**Pcdh20**

**Bcl11b**

**Supplementary Figure 6** Individual profile plots for the top ten annotated genes with the greatest absolute expression values detected in group P, which was dissected from zone P of the MCC tissue (black line) in relation to the overall expression profile plot for FCC and MCC zones (green background).

**Normalized Intensity Values**

**Normalized Intensity Values**

**Normalized Intensity Values**


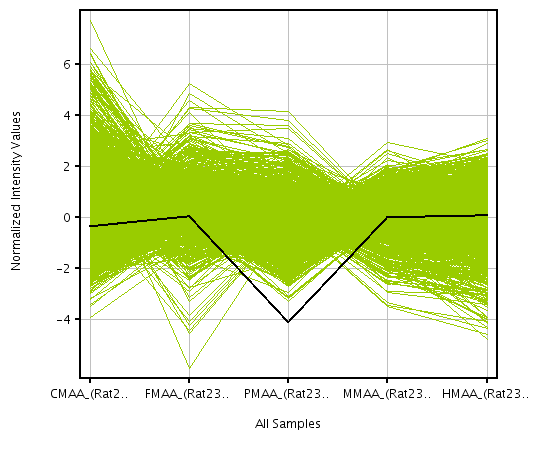

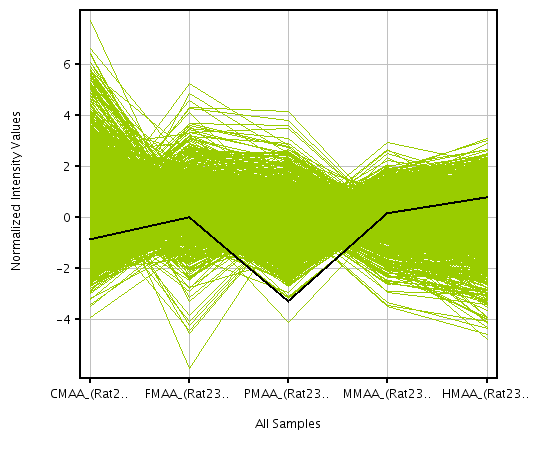

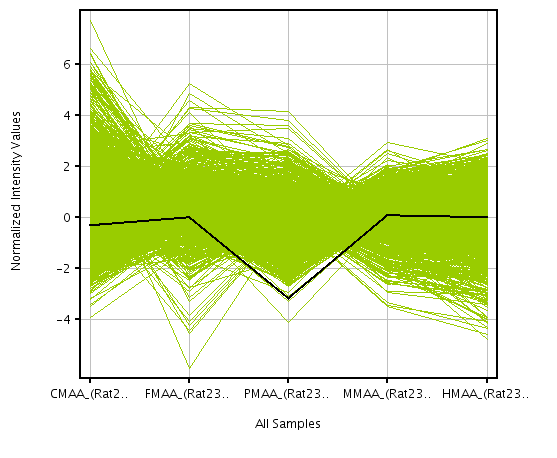

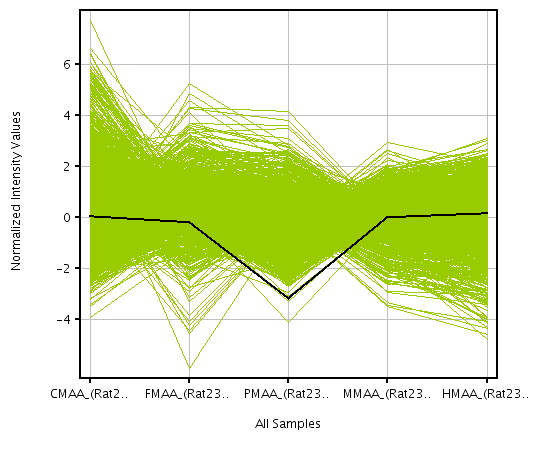

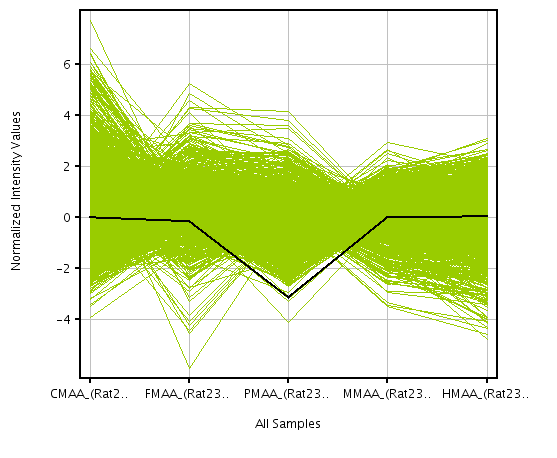

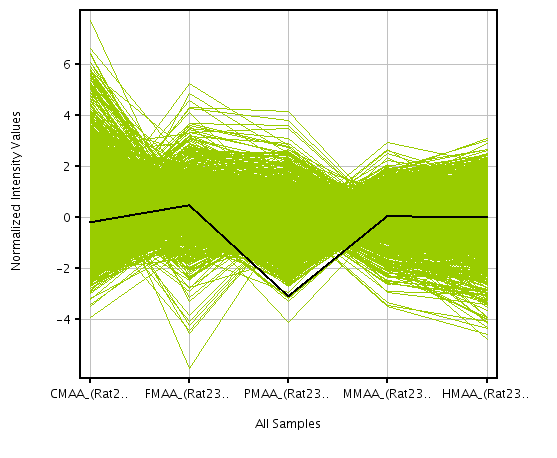

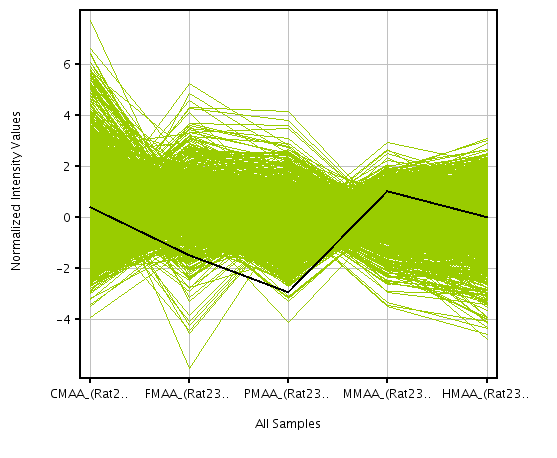

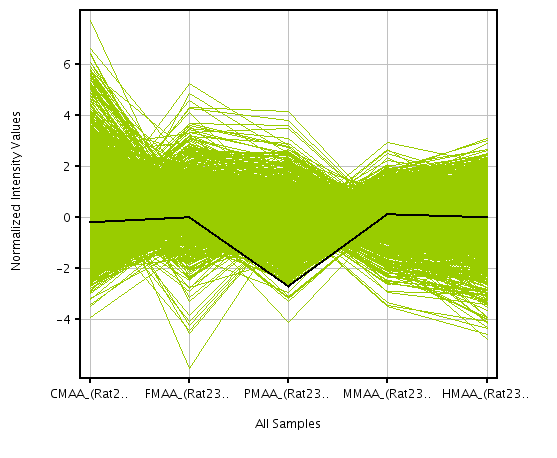

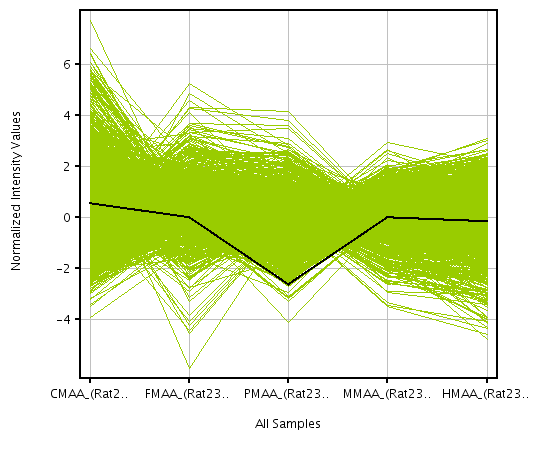

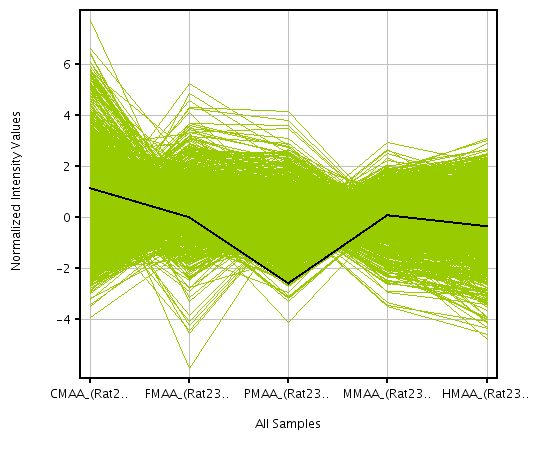


**Ubl5**

**Ccl9**

**Tp53**

**Hsp90ab1**

**Aplp2**

**Col4a2**

**Fgl2**

**Nab2**

**Kif13a**

**Atp11b**

**Supplementary Figure 7** Individual profile plots for the top ten annotated genes with the least absolute expression values detected in group P, which was dissected from zone P of the MCC tissue (black line) in relation to the overall expression profile plot for FCC and MCC zones (green background).


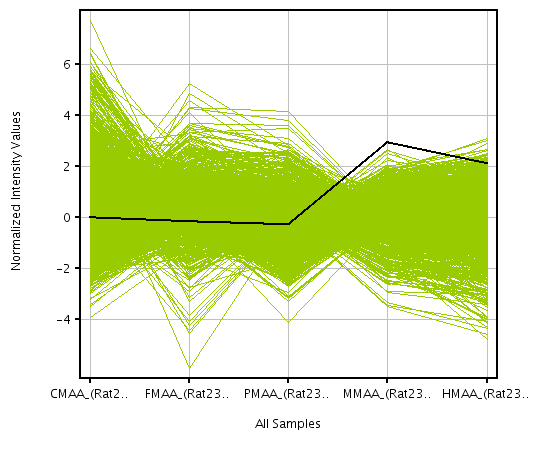

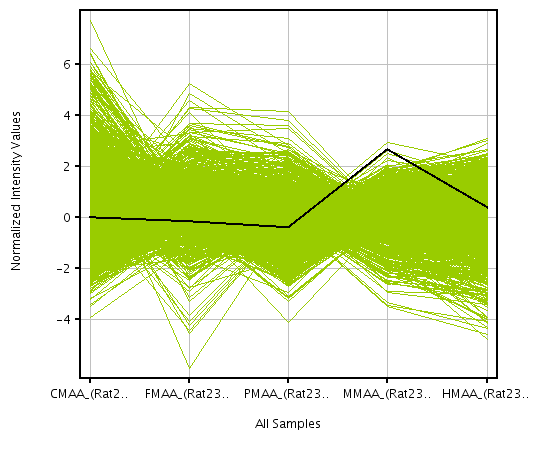

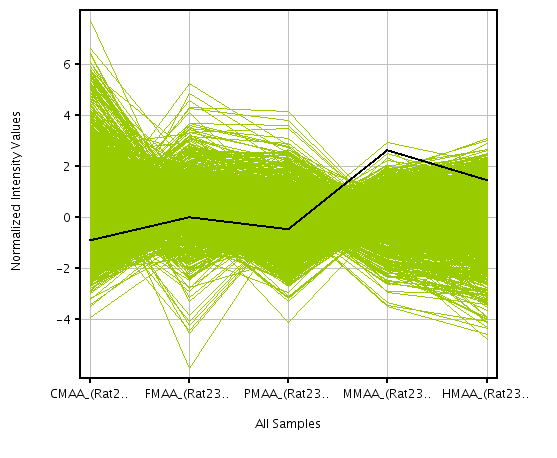

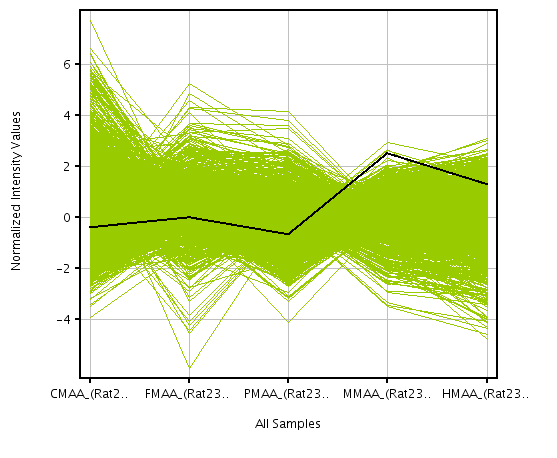

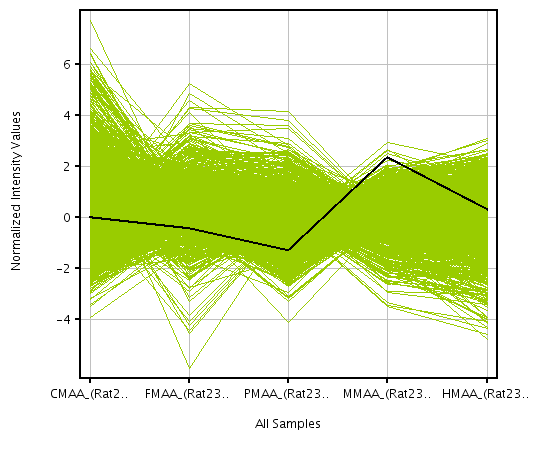

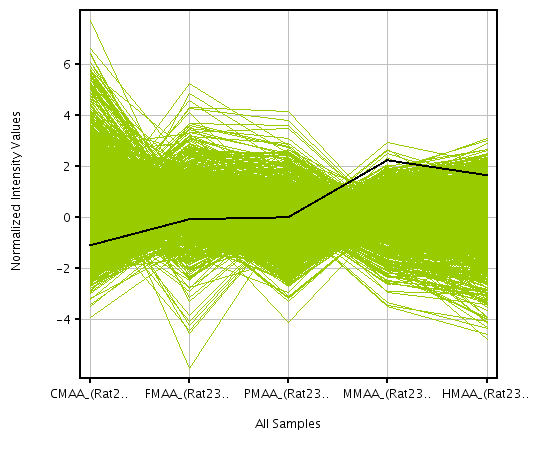

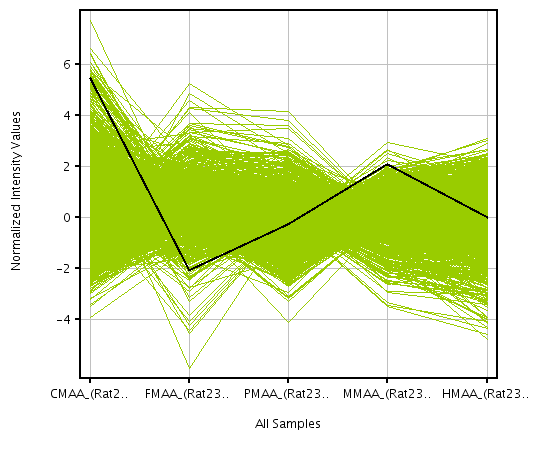

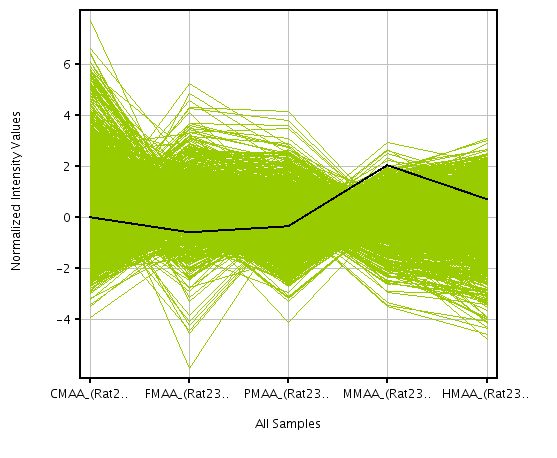

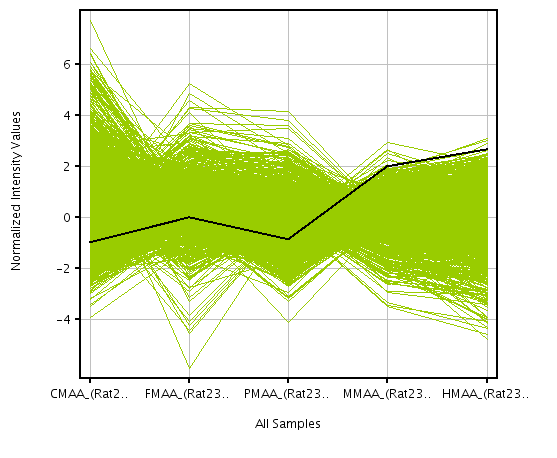

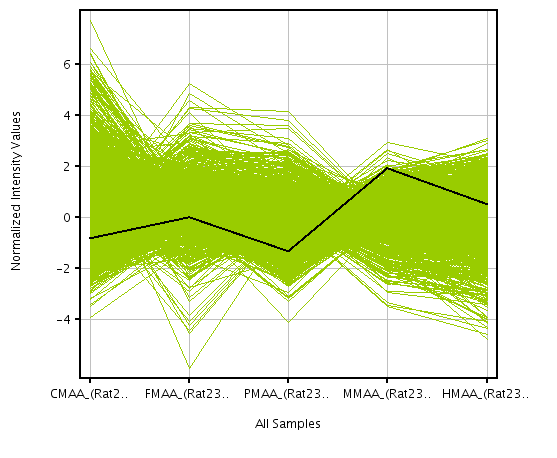


**Normalized Intensity Values**

**Normalized Intensity Values**

**Normalized Intensity Values**

**Serpinb10**

**Mmrn1**

**Plek**

**Pf4**

**Nubp2**

**Sstr2**

**Lect1**

**Treml1**

**RGD1564318**

**Pla2g2a**

**Supplementary Figure 8** Individual profile plots for the top ten annotated genes with the greatest absolute expression values detected in group M, which was dissected from zone M of the MCC tissue (black line) in relation to the overall expression profile plot for FCC and MCC zones (green background).


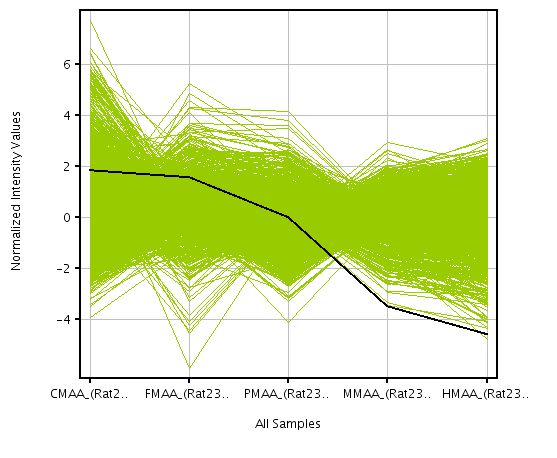

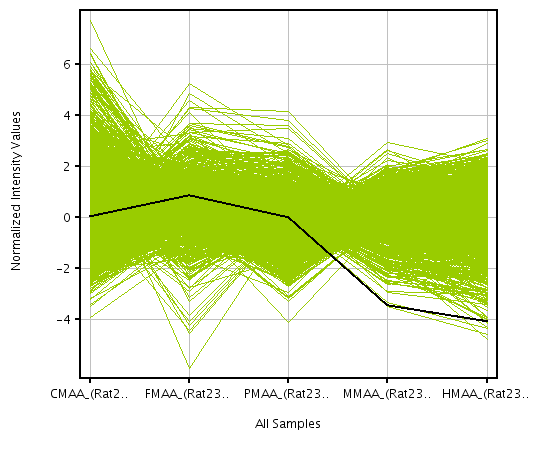

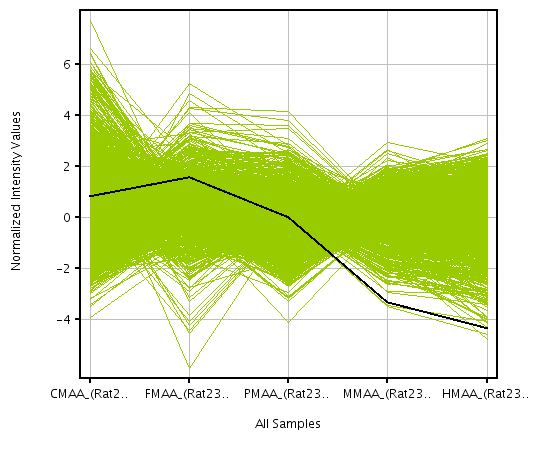

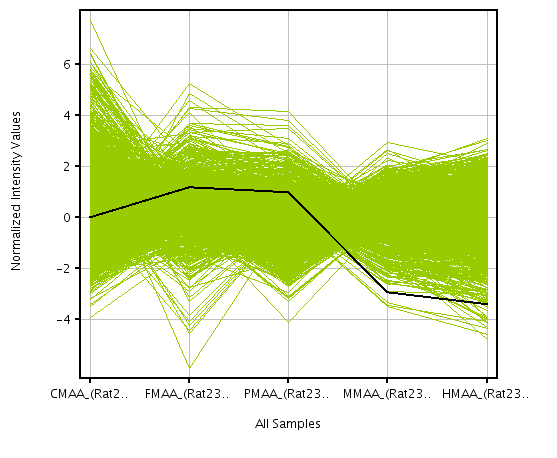

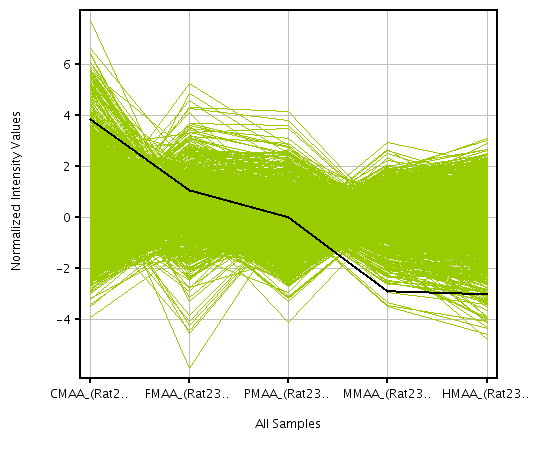

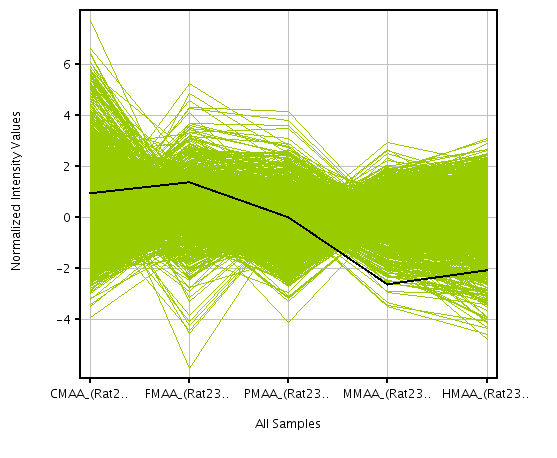

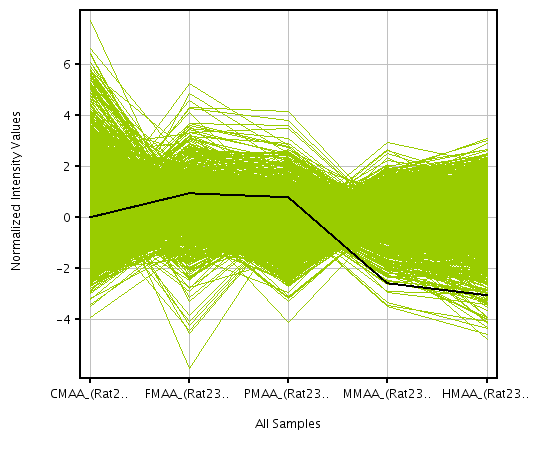

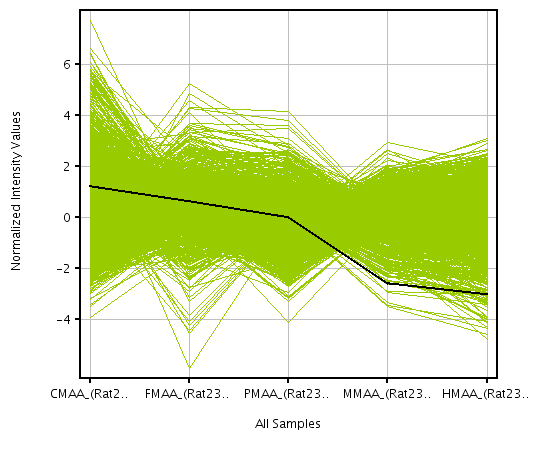

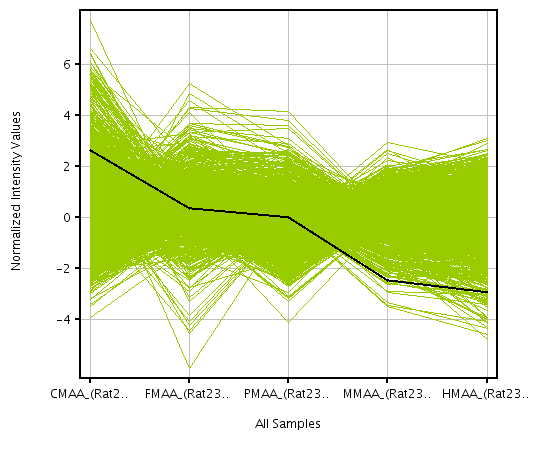

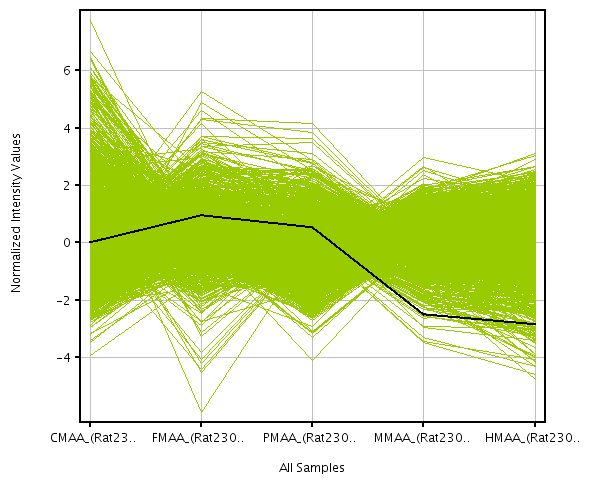


**Normalized Intensity Values**

**Normalized Intensity Values**

**Normalized Intensity Values**

**Angptl1**

**Cpxm2**

**Col14a1**

**Hmcn1**

**Itgbl1**

**Aoc3**

**Tspan2**

**Fibin**

**Pon3**

**Tspan2**

**Supplementary Figure 9** Individual profile plots for the top ten annotated genes with the least absolute expression values detected in group M, which was dissected from zone M of the MCC tissue (black line) in relation to the overall expression profile plot for FCC and MCC zones (green background).


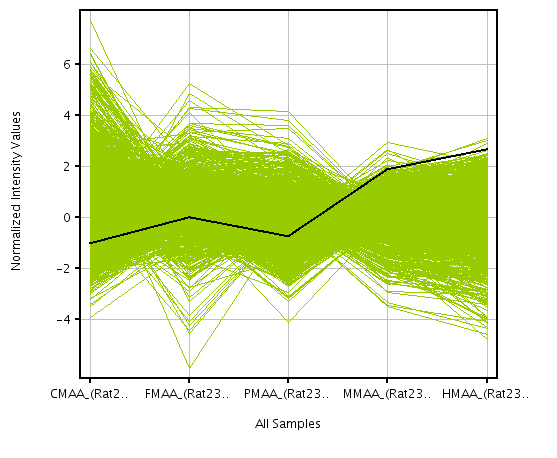

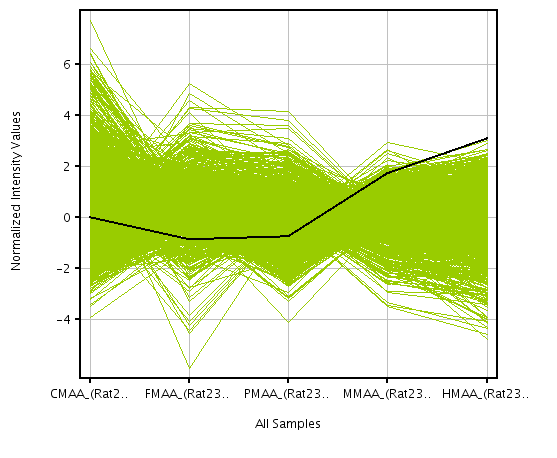

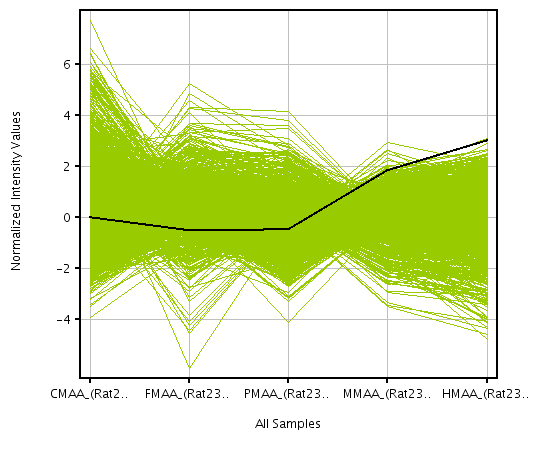

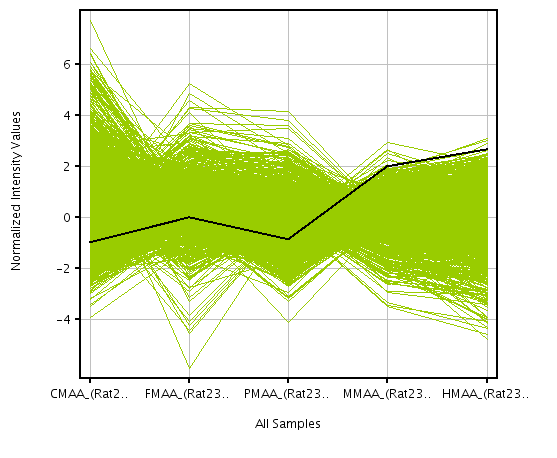

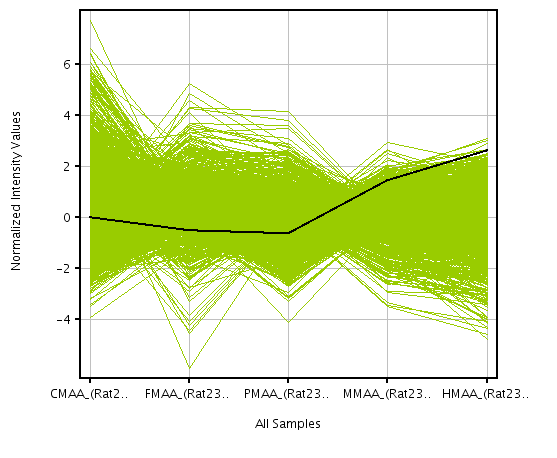

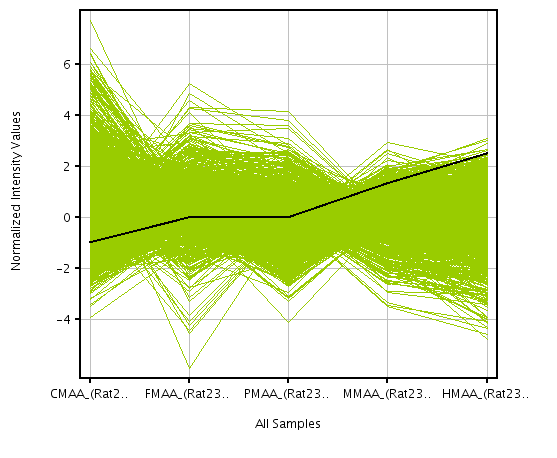

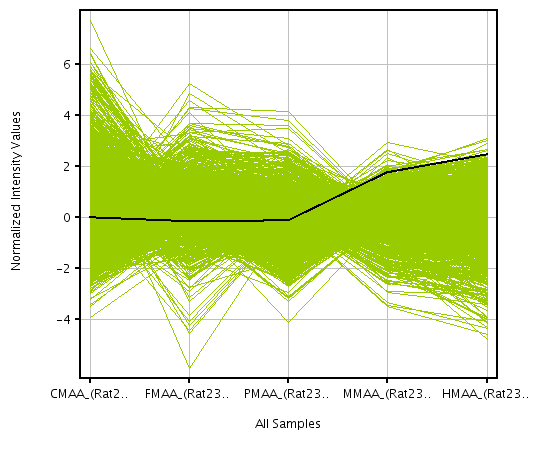

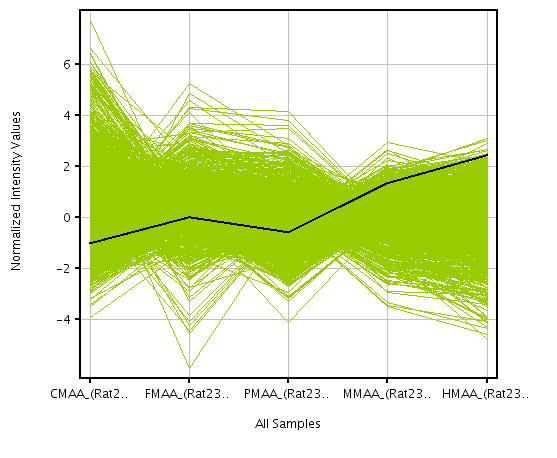

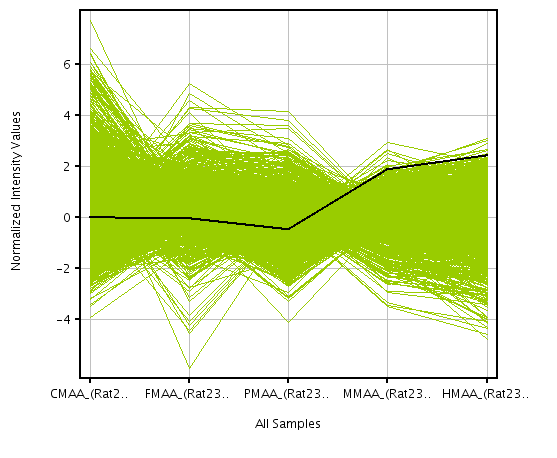

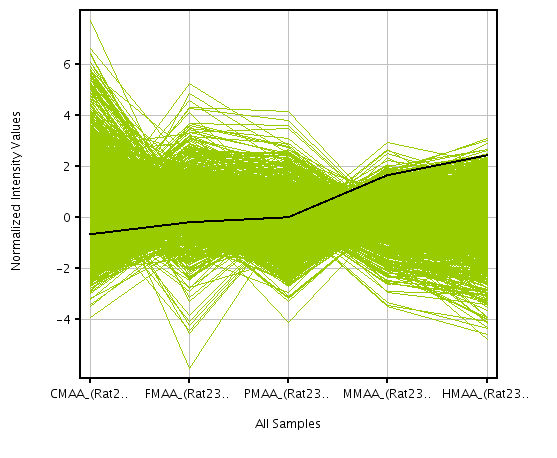


**Normalized Intensity Values**

**Normalized Intensity Values**

**Normalized Intensity Values**

**Car1**

**Slc4a1**

**RGD1564318**

**LOC100361706,**

**LOC682411, RGD1564318**

**Klf1**

**RGD1560020_predicted**

**Tal1**

**Cd3g**

**Cmah**

**Ctse**

**Supplementary Figure 10** Individual profile plots for the top ten annotated genes with the greatest absolute expression values detected in group H, which was dissected from zone H of the MCC tissue (black line) in relation to the overall expression profile plot for FCC and MCC zones (green background).


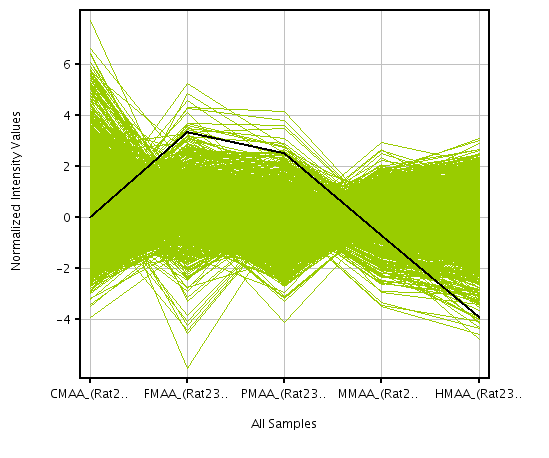

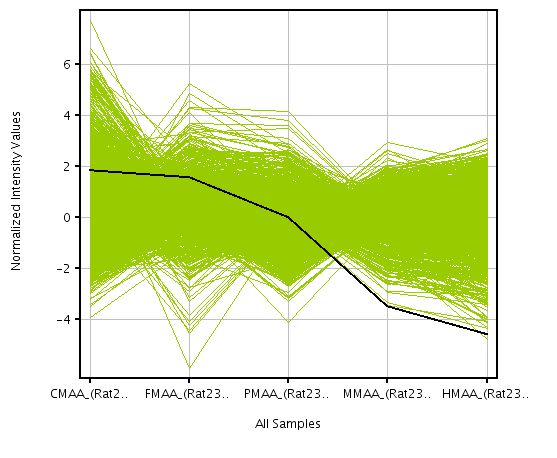

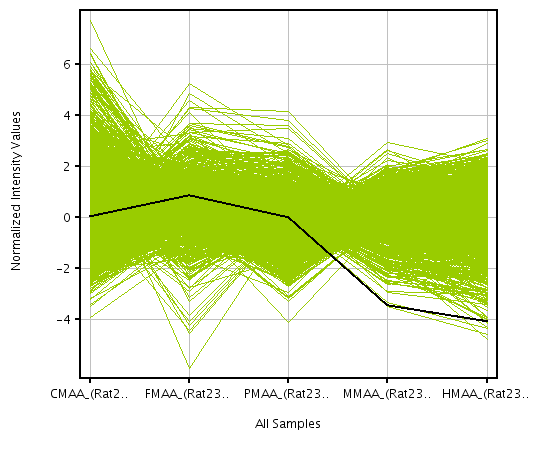

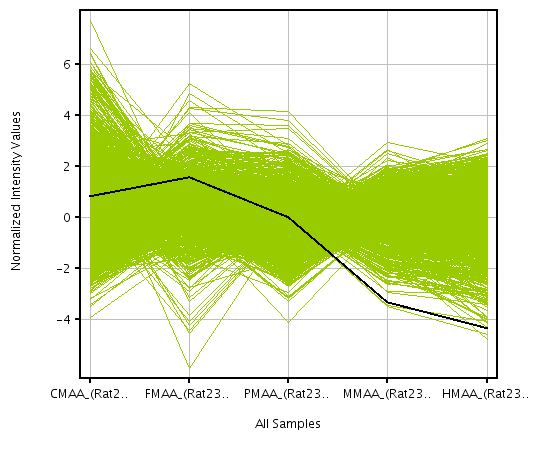

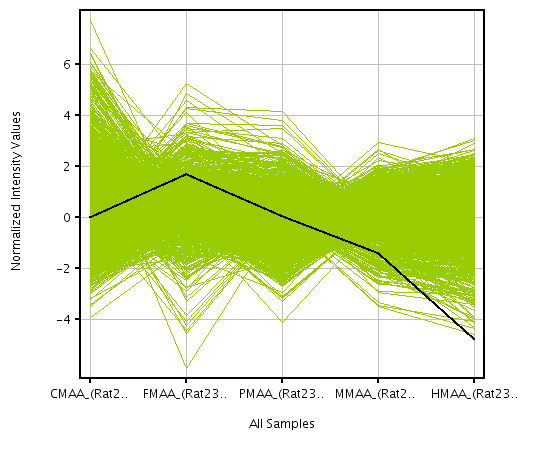

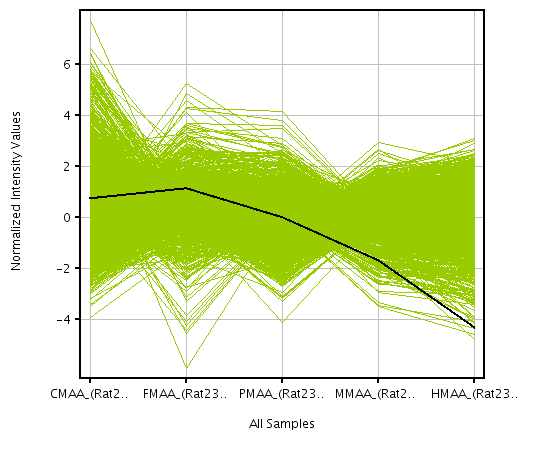

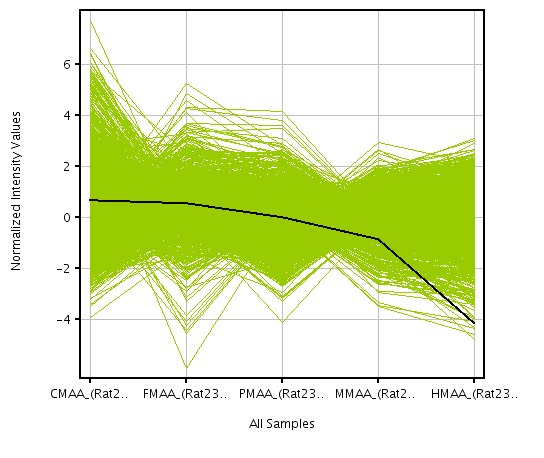

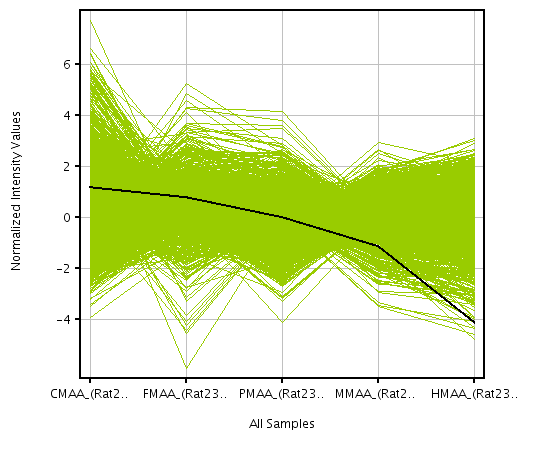

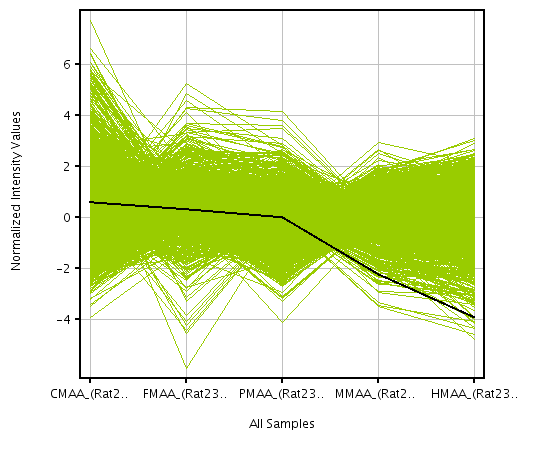

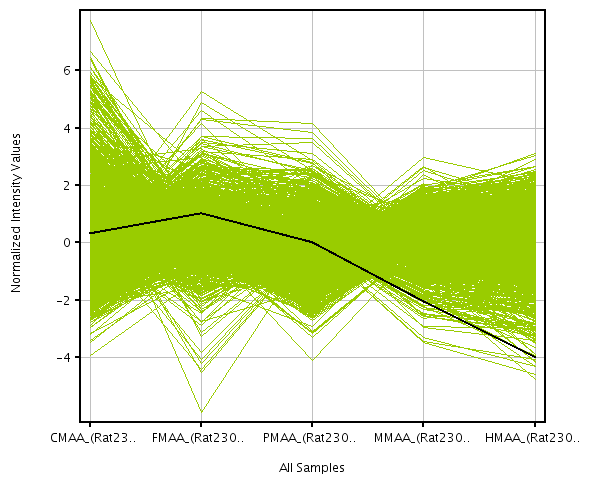


**Normalized Intensity Values**

**Normalized Intensity Values**

**Normalized Intensity Values**

**Fndc1**

**Angptl1**

**Cpxm2**

**Col14a1**

**Car9**

**Matn2**

**Fam180a**

**Fmod**

**Npas2**

**Cpxm2**

**Supplementary Figure 11** Individual profile plots for the top ten annotated genes with the least absolute expression values detected in group H, which was dissected from zone H of the MCC tissue (black line) in relation to the overall expression profile plot for FCC and MCC zones (green background).

**Supplementary Figure 12** Composite summary showing the identified canonical pathways by IPA analysis of the differentially expressed genes. When comparing MCC zones against FCC (four comparisons). The pathways are ranked by colored according to the z-score. A positive score indicates a significantly increased function (orange), a negative z-score indicates a significantly decreased function (blue), and an undetermined prediction is shown in gray color.
