# Supplementary material for: Implications of zonal architecture on differential gene expression profiling and altered pathway expressions in mandibular condylar cartilage
Source: Sci Rep. 2021 Aug 19;11:16915. doi: 10.1038/s41598-021-96071-7 (PMC8376865; doi:10.1038/s41598-021-96071-7)
Supplement: Supplementary file 3 — Supplementary Information 3. [file 41598_2021_96071_MOESM3_ESM.docx]

**Implications of zonal architecture on differential gene expression profiling and altered pathway expressions in mandibular condylar cartilage**

**Aisha M. Basudan^1,*^, Mohammad Azhar Aziz^2^ & Yanqi Yang^3^**

^1^ Division of Orthodontics, Dental Services Department, King Abdulaziz Medical City (KAMC) / King Abdullah International Medical Research Center (KAIMRC) / King Saud bin Abdulaziz University for Health Sciences (KSAU-HS), Ministry of National Guard-Health Affairs, Riyadh, 11426, Saudi Arabia.

^2^ King Abdullah International Medical Research Center (KAIMRC) / King Saud bin Abdulaziz University for Health Sciences (KSAU-HS), Colorectal Cancer Research Program, Ministry of National Guard-Health Affairs, Riyadh, 11426, Saudi Arabia.

^3^ Division of Paediatric Dentistry and Orthodontics, Faculty of Dentistry, The University of Hong Kong, 34 Hospital Road, Hong Kong SAR, China.

* Corresponding author A.M.B. (email: aisha_basudan@yahoo.com)


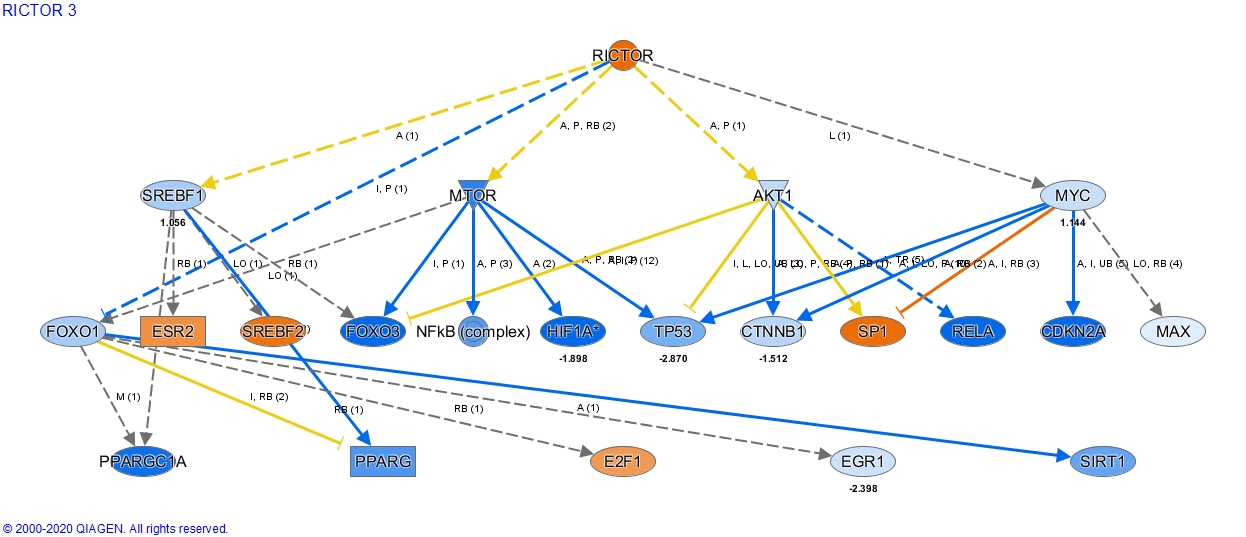


(a)


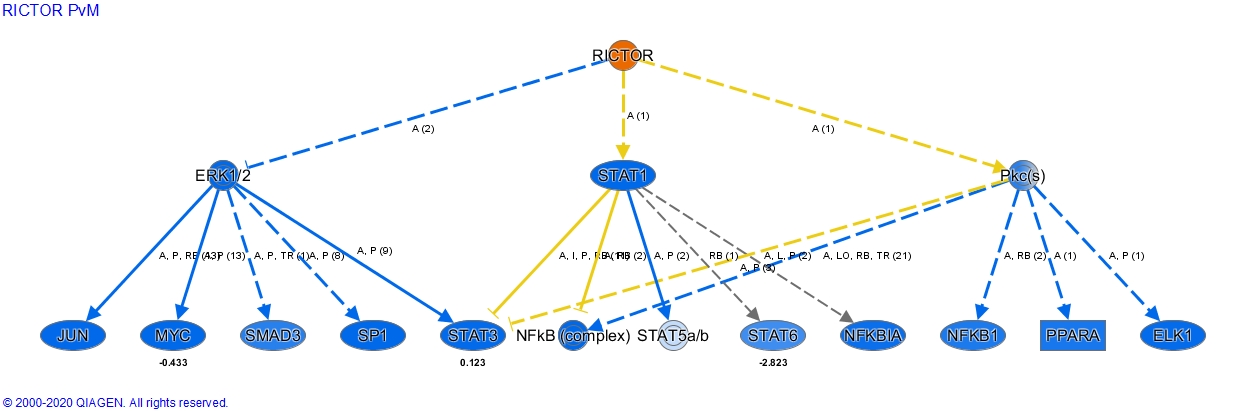


(b)


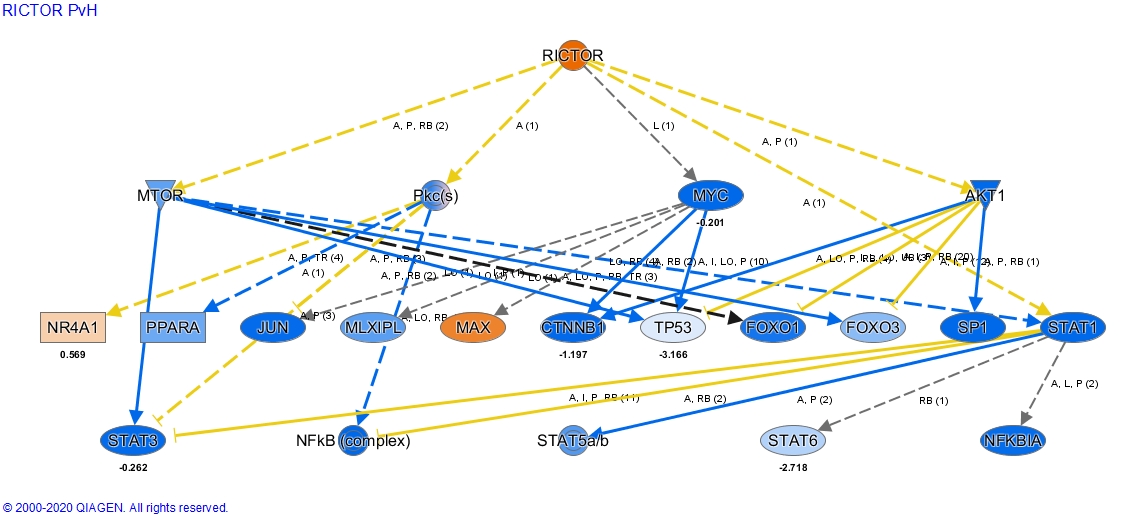


(c)

**
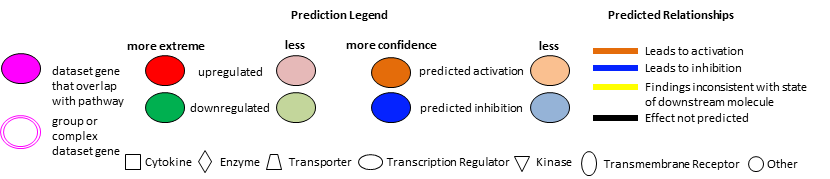
**

**Supplementary Figure 13** Mechanistic networks for RICTOR which is predicted to be among the top three activated upstream regulators in proliferative zone (PZ) when compared to femoral condylar cartilage; FCC (a), mature chondrocyte zone; MZ (a), and hypertrophic chondrocyte one; HZ (c). Upstream regulators interact with each other; in these networks it is postulated that RICTOR activate (orange color) or inhibit (blue color) other regulators to explain the gene expression changes observed in our data.

“Data were analyzed through the use of IPA (QIAGEN Inc., <https://www.qiagenbioinformatics.com/products/ingenuitypathway-analysis>).

Krämer, A., Green, J., Pollard, J. Jr. & Tugendreich, S. Causal analysis approaches in Ingenuity Pathway Analysis. *Bioinformatics*. **30**(4), 523-530 (2014).
