# Supplementary material for: Implications of zonal architecture on differential gene expression profiling and altered pathway expressions in mandibular condylar cartilage
Source: Sci Rep. 2021 Aug 19;11:16915. doi: 10.1038/s41598-021-96071-7 (PMC8376865; doi:10.1038/s41598-021-96071-7)

**Implications of zonal architecture on differential gene expression profiling and altered pathway expressions in mandibular condylar cartilage**

**Aisha M. Basudan^1,*^, Mohammad Azhar Aziz^2^ & Yanqi Yang^3^**

^1^ Division of Orthodontics, Dental Services Department, King Abdulaziz Medical City (KAMC) / King Abdullah International Medical Research Center (KAIMRC) / King Saud bin Abdulaziz University for Health Sciences (KSAU-HS), Ministry of National Guard-Health Affairs, Riyadh, 11426, Saudi Arabia.

^2^ King Abdullah International Medical Research Center (KAIMRC) / King Saud bin Abdulaziz University for Health Sciences (KSAU-HS), Colorectal Cancer Research Program, Ministry of National Guard-Health Affairs, Riyadh, 11426, Saudi Arabia.

^3^ Division of Paediatric Dentistry and Orthodontics, Faculty of Dentistry, The University of Hong Kong, 34 Hospital Road, Hong Kong SAR, China.

* Corresponding author A.M.B. (email: aisha_basudan@yahoo.com)

**Normalized Intensity Values**

**RGD1311447/GAPDH**


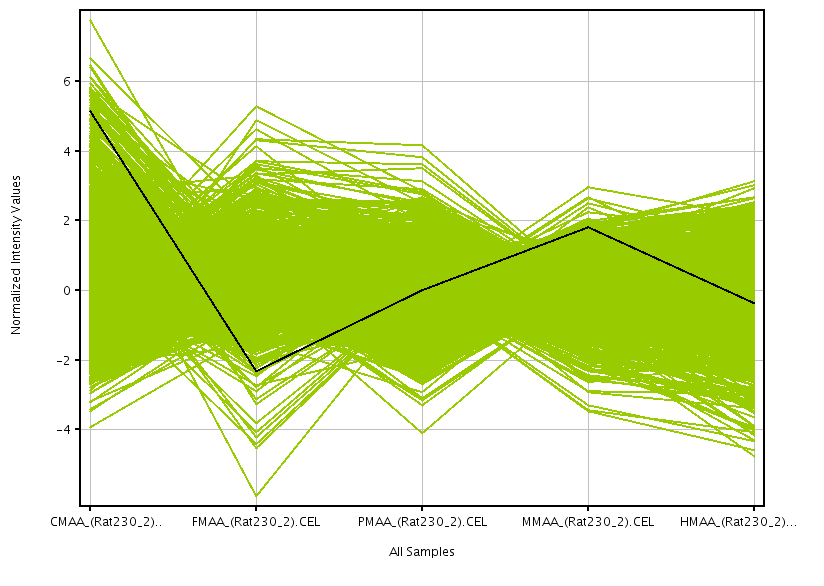


**RGD1311447**

**C**

**PZ**

**MZ**

**FZ**

**HZ**

**(a)**


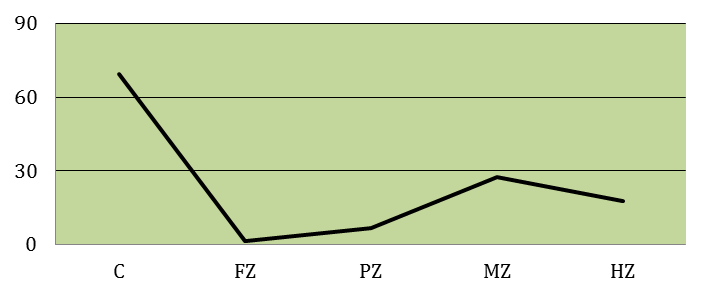


**(b)**

**Supplementary Figure 14** Validation of MAA results by RT-qPCR. **(a)** Profile plot of MAA data for RGD1311447 gene expression (black line) as determined in the FCC tissue (C), which is the control, and in the fibroblastic (FZ), proliferative (PZ), mature (MZ), and hypertrophic (HZ) zones of the MCC tissue. **(b)** Gene expressions for RGD1311447 using qRT-PCR presented as a ratio of gene expression to that of GAPDH*.*


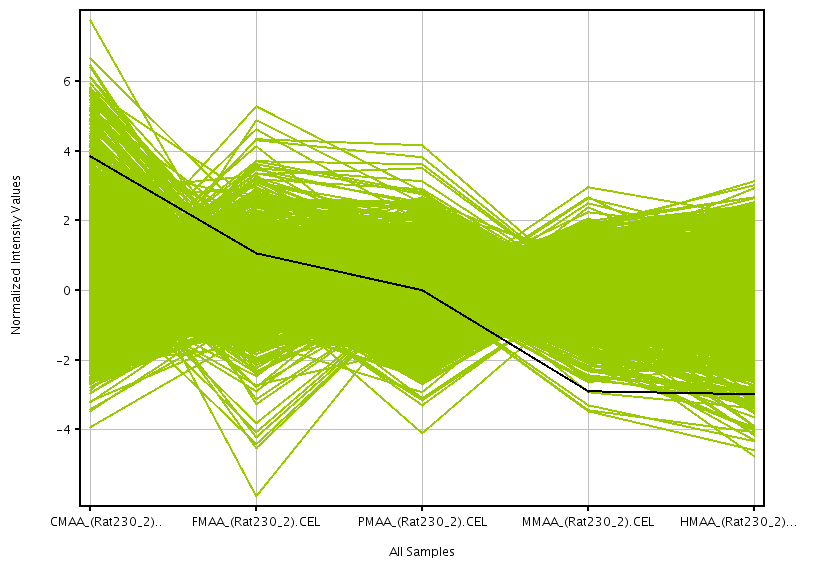


**Normalized Intensity Values**

**Itgbl1/GAPDH**

**(a)**

**(b)**

**C**

**PZ**

**MZ**

**FZ**

**HZ**

**Itgbl1**

**Supplementary Figure 15** Validation of MAA results by RT-qPCR. **(a)** Profile plot of MAA data for Itgbl1 gene expression (black line) as determined in the FCC tissue (C), which is the control, and in the fibroblastic (FZ), proliferative (PZ), mature (MZ), and hypertrophic (HZ) zones of the MCC tissue. **(b)** Gene expressions for Itgbl1 using qRT-PCR presented as a ratio of gene expression to that of GAPDH*.*


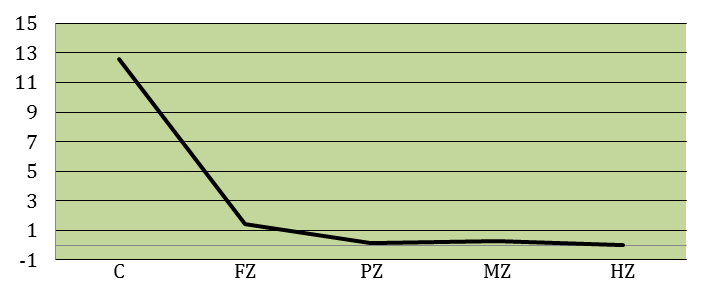

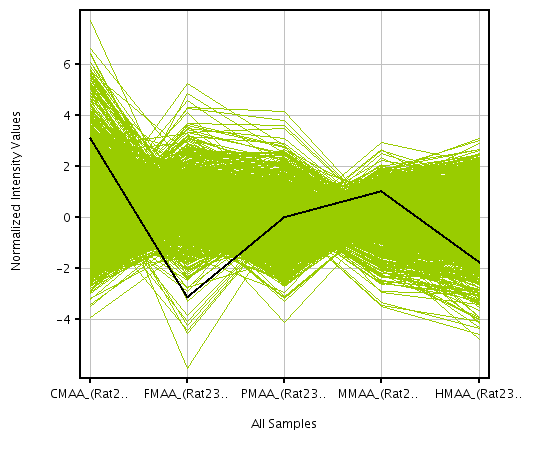


**Normalized Intensity Values**

**(a)**

**(b)**

**C**

**PZ**

**MZ**

**FZ**

**HZ**

**Supplementary Figure 16** Validation of MAA results by RT-qPCR. **(a)** Profile plot of MAA data for Cmtm5 gene expression (black line) as determined in the FCC tissue (C), which is the control, and in the fibroblastic (FZ), proliferative (PZ), mature (MZ), and hypertrophic (HZ) zones of the MCC tissue. **(b)** Gene expressions for Cmtm5 using qRT-PCR presented as a ratio of gene expression to that of GAPDH*.*

**Cmtm5/GAPDH**

**Cmtm5**


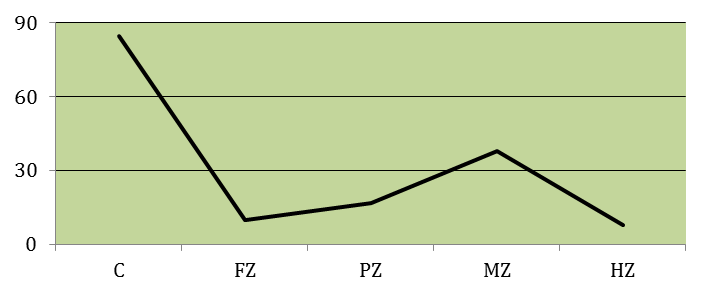

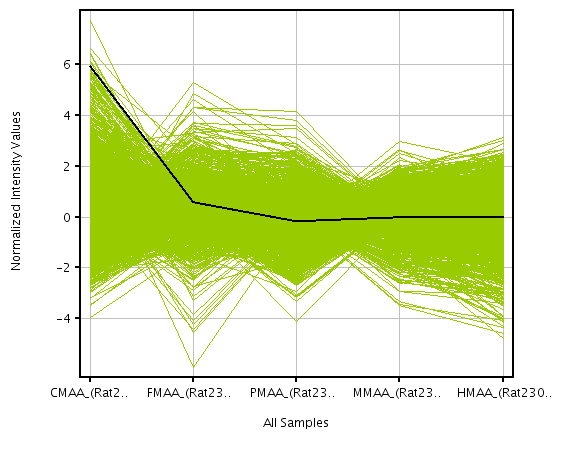


**Normalized Intensity Values**

**(a)**

**(b)**

**C**

**PZ**

**MZ**

**FZ**

**HZ**

**Supplementary Figure 17** Validation of MAA results by RT-qPCR. **(a)** Profile plot of MAA data for Gdf10 gene expression (black line) as determined in the FCC tissue (C), which is the control, and in the fibroblastic (FZ), proliferative (PZ), mature (MZ), and hypertrophic (HZ) zones of the MCC tissue. **(b)** Gene expressions for Gdf10 using qRT-PCR presented as a ratio of gene expression to that of GAPDH*.*

**Gdf10/GAPDH**

**Gdf10**


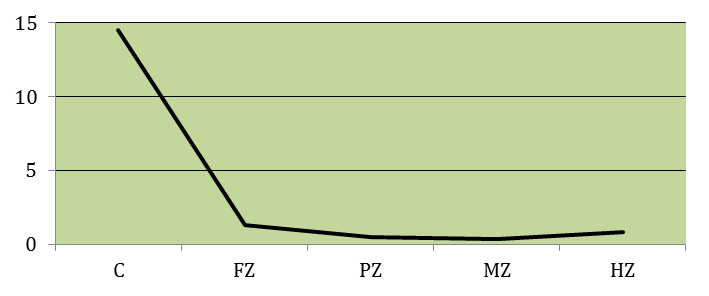

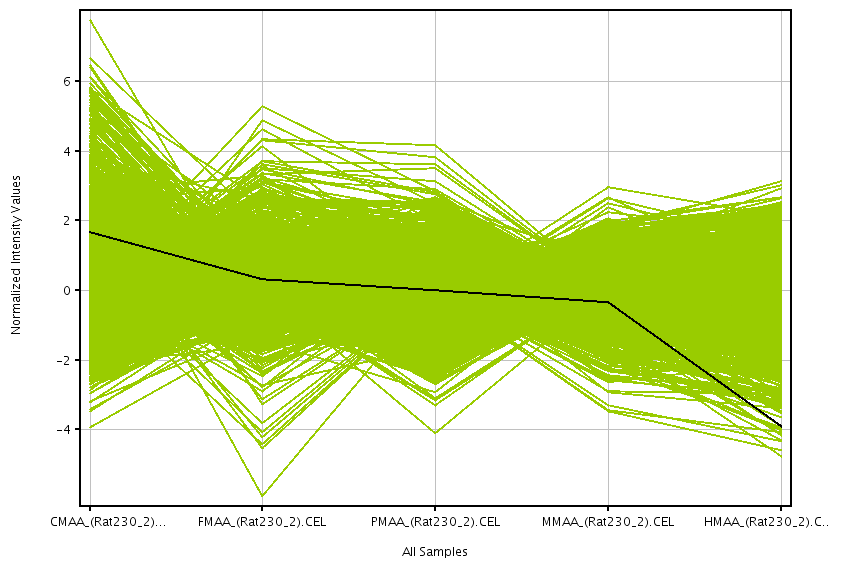


**Ucma**

**Normalized Intensity Values**

**Ucma/GAPDH**

**(a)**

**(b)**

**C**

**PZ**

**MZ**

**FZ**

**HZ**

**Supplementary Figure 18** Validation of MAA results by RT-qPCR. **(a)** Profile plot of MAA data for Ucma gene expression (black line) as determined in the FCC tissue (C), which is the control, and in the fibroblastic (FZ), proliferative (PZ), mature (MZ), and hypertrophic (HZ) zones of the MCC tissue. **(b)** Gene expressions for Ucma using qRT-PCR presented as a ratio of gene expression to that of GAPDH*.*


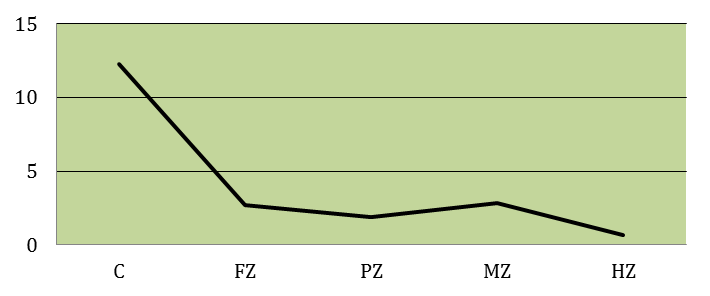


**(a)**

**Normalized Intensity Values**

**Ctsz/GAPDH**

**(b)**

**Supplementary Figure 19** Validation of MAA results by RT-qPCR. **(a)** Profile plot of MAA data for Ctsz gene expression (black line) as determined in the FCC tissue (C), which is the control, and in the fibroblastic (FZ), proliferative (PZ), mature (MZ), and hypertrophic (HZ) zones of the MCC tissue. **(b)** Gene expressions for Ctsz using qRT-PCR presented as a ratio of gene expression to that of GAPDH*.*


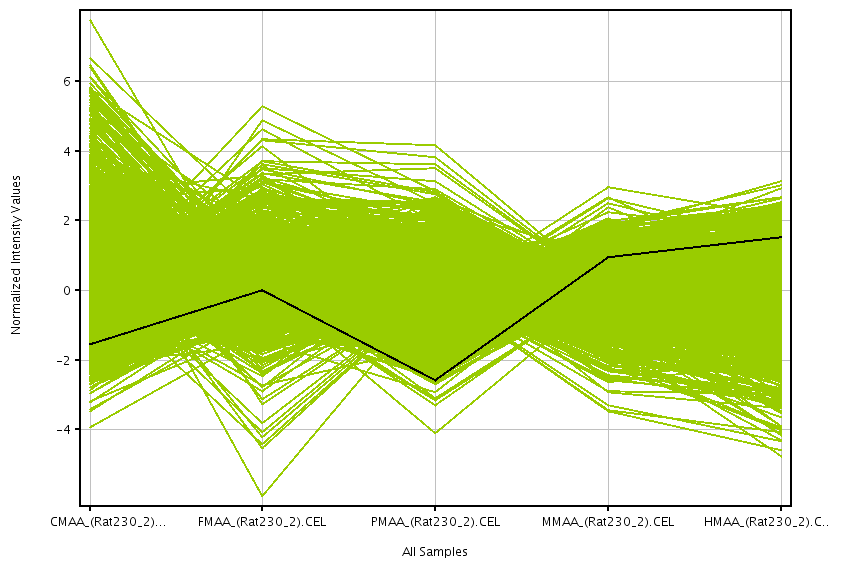


**Ctsz**


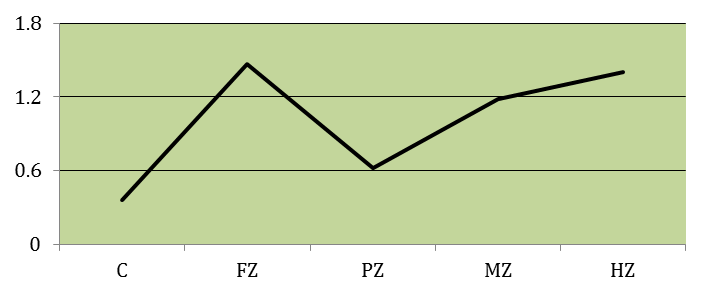


**C**

**PZ**

**MZ**

**FZ**

**HZ**


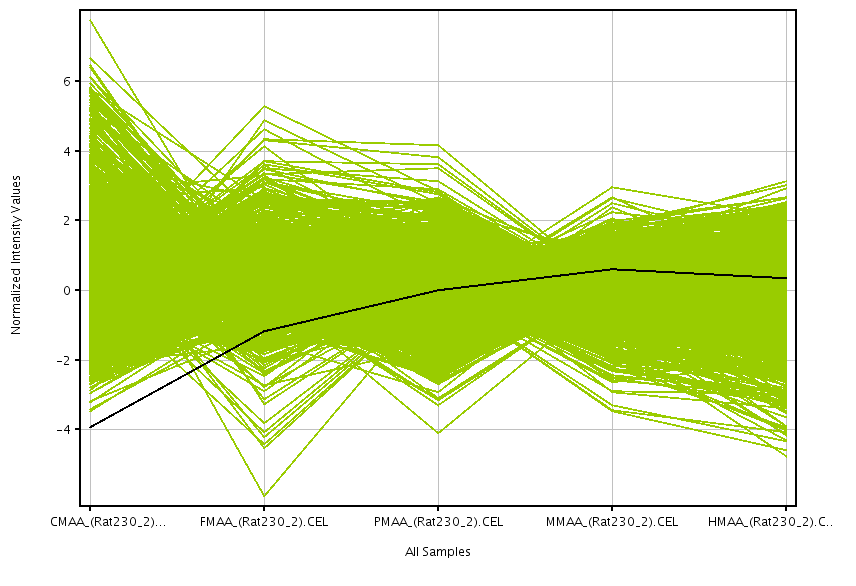


**Normalized Intensity Values**

**(a)**

**(b)**

**C**

**PZ**

**MZ**

**FZ**

**HZ**

**Supplementary Figure 20** Validation of MAA results by RT-qPCR. **(a)** Profile plot of MAA data for Dusp27 gene expression (black line) as determined in the FCC tissue (C), which is the control, and in the fibroblastic (FZ), proliferative (PZ), mature (MZ), and hypertrophic (HZ) zones of the MCC tissue. **(b)** Gene expressions for Dusp27 using qRT-PCR presented as a ratio of gene expression to that of GAPDH*.*

**Dusp27/GAPDH**

**Dusp27**


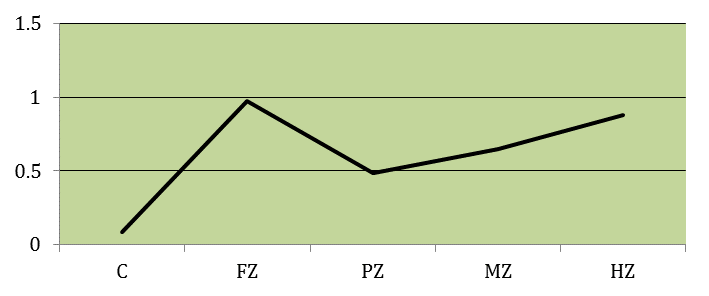


**Normalized Intensity Values**

**(a)**

**(b)**

**C**

**PZ**

**MZ**

**FZ**

**HZ**

**Supplementary Figure 21** Validation of MAA results by RT-qPCR. **(a)** Profile plot of MAA data for Car9 gene expression (black line) as determined in the FCC tissue (C), which is the control, and in the fibroblastic (FZ), proliferative (PZ), mature (MZ), and hypertrophic (HZ) zones of the MCC tissue. **(b)** Gene expressions for Car9 using qRT-PCR presented as a ratio of gene expression to that of GAPDH*.*

**Car9/GAPDH**


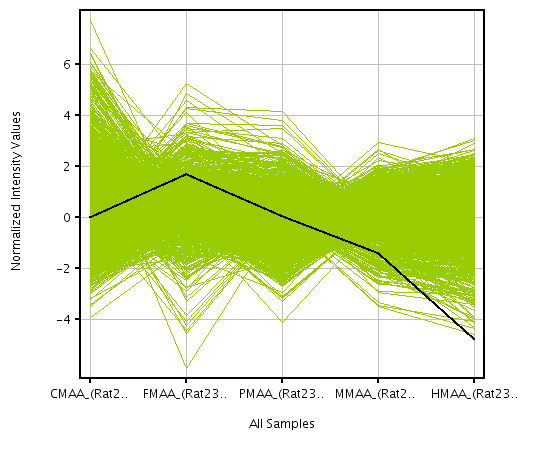


**Car9**


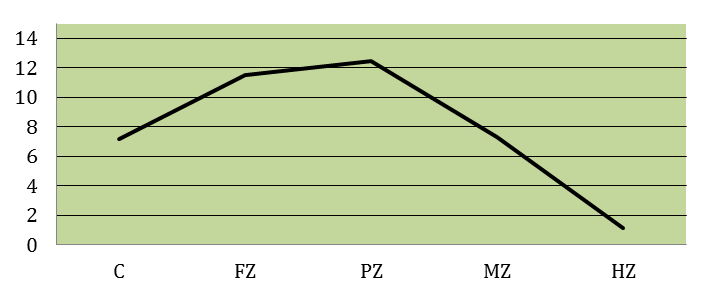


**Normalized Intensity Values**

**(a)**

**(b)**

**C**

**PZ**

**MZ**

**FZ**

**HZ**

**Supplementary Figure 22** Validation of MAA results by RT-qPCR. **(a)** Profile plot of MAA data for Drd4 gene expression (black line) as determined in the FCC tissue (C), which is the control, and in the fibroblastic (FZ), proliferative (PZ), mature (MZ), and hypertrophic (HZ) zones of the MCC tissue. **(b)** Gene expressions for Drd4 using qRT-PCR presented as a ratio of gene expression to that of GAPDH*.*

**Drd4/GAPDH**


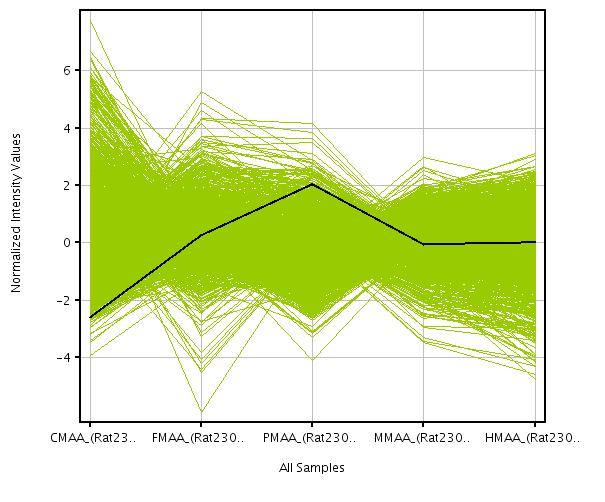


**Drd4**


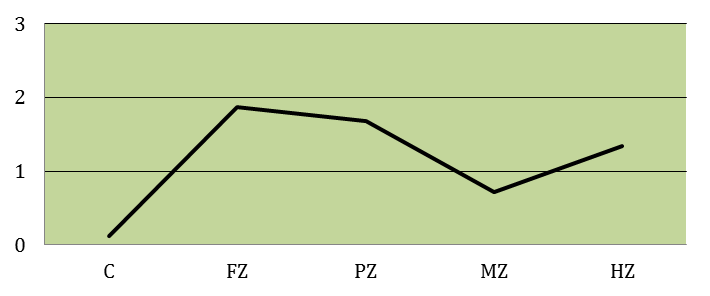


**Normalized Intensity Values**


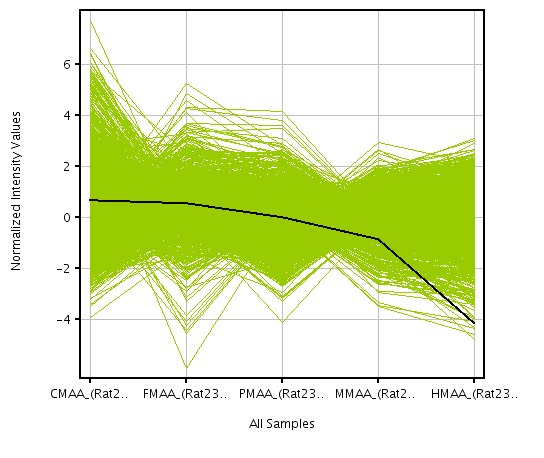


**(a)**

**(b)**

**C**

**PZ**

**MZ**

**FZ**

**HZ**

**Supplementary Figure 23** Validation of MAA results by RT-qPCR. **(a)** Profile plot of MAA data for Fam180a gene expression (black line) as determined in the FCC tissue (C), which is the control, and in the fibroblastic (FZ), proliferative (PZ), mature (MZ), and hypertrophic (HZ) zones of the MCC tissue. **(b)** Gene expressions for Fam180a using qRT-PCR presented as a ratio of gene expression to that of GAPDH*.*

**Fam180a/GAPDH**

**Fam180a**


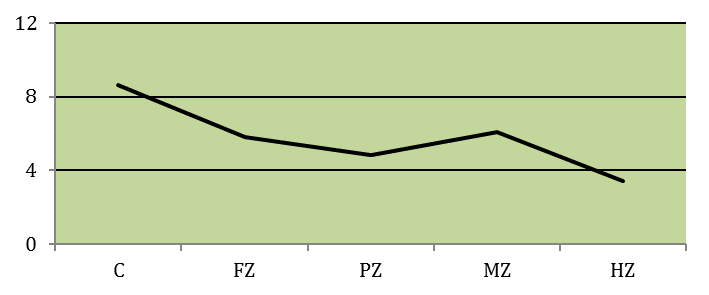

Supplement: Supplementary file 4 — Supplementary Information 4. [file 41598_2021_96071_MOESM4_ESM.docx]
